# Air Pollution and Disrupted Microbiomes: Tracing the Impact on Human Health

**DOI:** 10.7759/cureus.89267

**Published:** 2025-08-02

**Authors:** Treesa Thomas, Abdulqadir J Nashwan

**Affiliations:** 1 Nursing and Midwifery Research Department, Hamad Medical Corporation, Doha, QAT

**Keywords:** air-borne toxins, air pollutants, air pollution, dysbiosis, gut-brain axis, gut-lung axis, gut microbiota, particulate matter, pm2.5

## Abstract

Air pollution has been linked to various illnesses; however, recent research suggests that it may also impact the gut microbiota, which is crucial to human health. This scoping review aims to synthesize the existing literature on the impact of air pollution on gut microbiota and its associated health consequences. A comprehensive search was conducted across two databases, PubMed and Scopus, resulting in the selection of 159 papers for in-depth analysis. This review examines 158 research studies published between 2010 and 2025, investigating the impact of air pollution on gut flora. Recent studies on pollutants such as PM2.5, heavy metals, and volatile organic compounds (VOCs) have shown an increasing interest in the topic. Air pollution continuously alters the composition of the gut microbiota, which in turn impacts respiratory, neurological, cardiovascular, and metabolic health. The gut-lung and gut-brain axes play essential roles in mediating these effects. The impact of air pollution can be mitigated with protective measures, including probiotics, symbiotics, and dietary adjustments. The health impacts of early and prolonged exposure to air pollution are long-lasting, especially for children. Air pollution is increasingly influencing gut microbiota and can potentially exacerbate several illnesses. Further investigations are necessary to elucidate the underlying mechanisms and the broader public health implications.

## Introduction and background

Air pollution is the fourth greatest health concern in the world, causing an estimated 6.7 million deaths per year [1]. Furthermore, according to the Global Burden of Disease (GBD) report, fine particulate matter (PM2.5) is the fifth-leading global risk factor for mortality, contributing to approximately 4.2 million preventable deaths annually [2]. Air pollution is a complex blend of various substances, encompassing gases such as carbon dioxide, carbon monoxide, ozone, nitric oxide, sulfur dioxide, and volatile organic compounds (VOCs), as well as PM and benzene [3]. Approximately three billion people worldwide are known to be exposed to household air pollution (HAP) from the use of solid cooking fuels, which poses a significant health risk. Particulate matter with a diameter of less than 2.5 μm (PM2.5), a known health risk, exposes people who live in homes that largely utilize solid fuels (coal, charcoal, wood, crop wastes, and dung) for cooking to high amounts of HAP [4].

Humans are regarded as superorganisms, as symbiotic cells and microbes. Without question, microorganisms are important to human existence. These beneficial microorganisms, collectively known as microbiota, are essential for bodily processes and can lead to severe infections. Specifically, a significant portion of the human microbiome consists of microorganisms. These helpful, innocuous, and occasionally opportunistic microorganisms are typical components of the human body's microbial flora [5].

The long-term effects of environmental exposures on the gut microbiota are poorly understood, even though variables such as diet, pathogen infections, and antibiotic use can rapidly alter microbial composition, leading to dysbiosis and disrupting host-microbiome interactions. A recent study has identified air pollution as a significant contributor to microbial imbalances. Even short-term exposure to air pollution has been shown to significantly alter microbial interactions, potentially leading to dysbiosis [5,6]. The mucociliary route carries large quantities of inhaled PM, which is quickly removed from the lung's alveolar cells before being sent to the intestine. Ingested PM initiated the dysbiosis of gut bacteria, which was caused by gastrointestinal inflammation. Through the gut-brain axis, gut microbial dysbiosis contributes to various health issues, including mental health problems. Through the vagus nerve, gut bacteria can affect the central nervous system. The gut is exposed to air contaminants, such as PM and heavy metals, through ingestion or inhalation. Short-chain fatty acids (SCFAs), necessary for brain-gut neurotransmitter signaling, are gut microbiota-associated metabolites that are further disrupted or altered by PMs-induced gut dysbiosis [5].

The use of fruit-derived biomolecules with immunostimulant and antioxidative properties [7], fish by-product proteins that support a healthy complexion [8], the probiotic potential of *Bacillus megaterium* Renuspore® for gut health and detoxification [9], *Lactiplantibacillus plantarum* in reducing PM-associated pulmonary inflammation [9], and the use of prebiotics, probiotics, and synbiotics to improve gut microbiota and permeability in children exposed to air pollution [10] are all promising ways to maintain a healthy gut microbiome and mitigate the negative effects of air pollution on human health.

In this scoping review, we aimed to investigate the interplay between air pollution, gut microbiota, and their associated health issues in humans.

## Review

Methods

Rationale

This review was conducted to compile published or publicly accessible data on the interplay between air pollution and gut microbiota, as well as their impact on human health. This scoping review adheres to the guidelines outlined in the Preferred Reporting Items for Systematic Reviews and Meta‐Analyses extension for Scoping Reviews (PRISMA‐ScR) checklist [11].

Protocol Registration

No formal review protocol was registered for this scoping review.

Eligibility Criteria

Studies were included in the review based on the following criteria: publications had to be in English, with no restrictions regarding the year of publication. Both original research and review articles were considered eligible. However, short communications such as editorials, letters to the editor, commentaries, and grey literature were excluded to maintain a focus on peer-reviewed scientific evidence.

Search Strategies

An extensive search was conducted from January 16 to 25, 2025, across two electronic databases: PubMed and Scopus. Table 1 shows the search terms utilized. To guarantee the retrieval of all pertinent studies based on their indexed words in the available databases, Medical Subject Headings (MeSH) phrases were included where appropriate.

**Table 1:** Search terms used in databases.

Category	Term
Air Pollution	“Air pollution” OR “Air pollutants” OR “Particulate matters” OR “PM2.5” OR “Environment pollutants” OR “Air-borne toxins”
AND Gut microbiota	“Gut microbiota” OR “Gut microbiome” OR “Gastrointestinal microbiota” OR “Dysbiosis”

Data Filtration Process

The selection of studies followed a three-stage screening process conducted by two independent reviewers. Initially, articles were screened based on their titles to exclude those that were irrelevant. The second stage involved reviewing abstracts to determine potential relevance. Finally, full-text articles were assessed for eligibility according to the inclusion criteria. At each step, any disagreements between the two reviewers were resolved through discussion or by consulting a third reviewer to reach a consensus. This rigorous process aimed to enhance the reliability and consistency of the study selection.

Data extraction was conducted independently by two reviewers (AJN and TT) to ensure accuracy and minimize bias. The process focused on capturing both general and outcome-specific information from each included study. General information encompassed details such as the first author’s name, country of origin, year of publication, the methodological approach used, and a summary of the main findings. In addition, the reviewers extracted the primary outcomes of each study, with particular attention to the key results relevant to the relationship between air pollution, gut microbiota, and health outcomes. This dual-layered extraction approach ensured a structured synthesis of evidence while maintaining the integrity of individual study contributions.

Results

**Figure 1:**
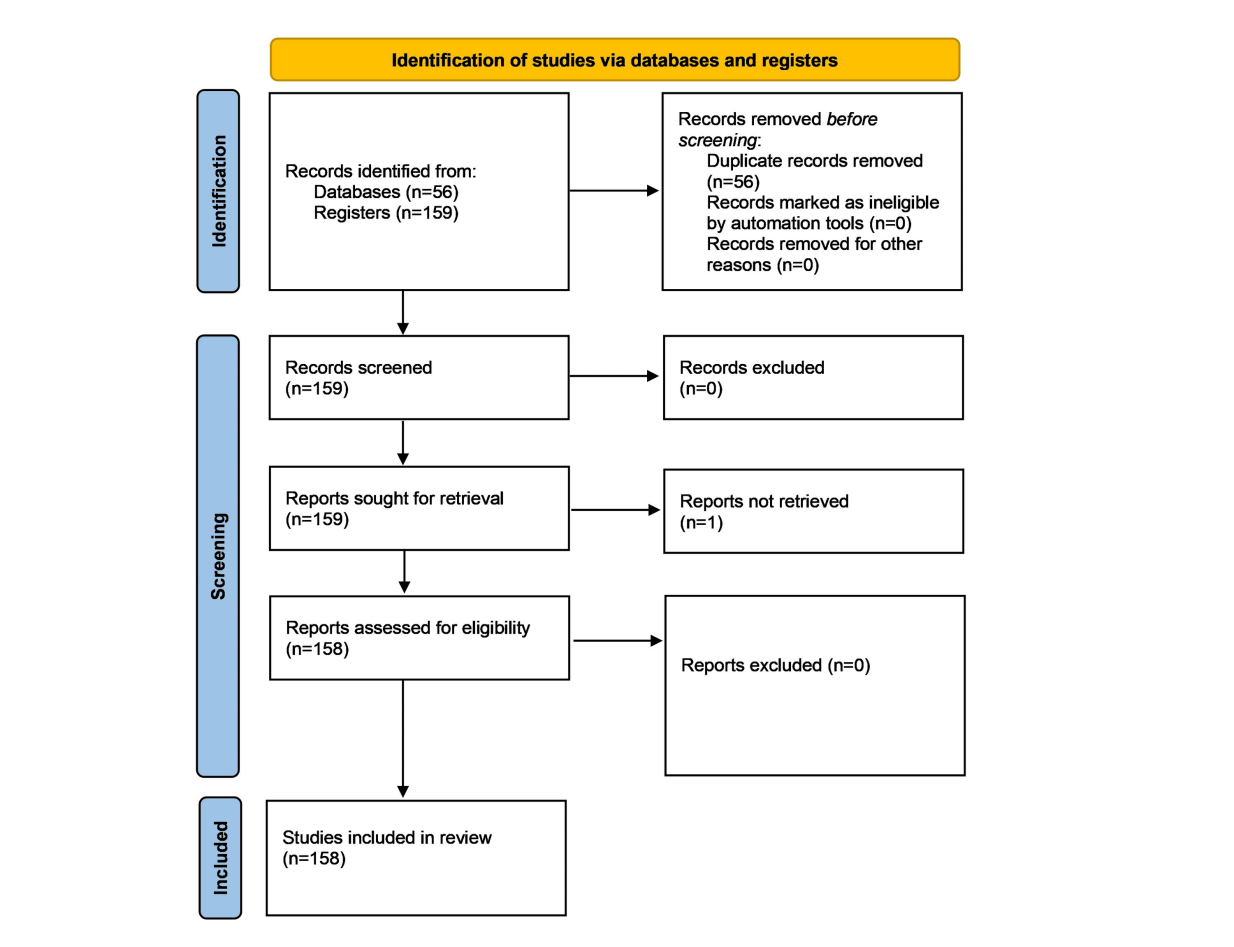
PRISMA‐ScR chart summarizing the filtration process. PRISMA‐ScR: Preferred Reporting Items for Systematic Reviews and Meta‐Analyses extension for Scoping Reviews

Our search yielded 158 studies, comprising 82 original articles, 68 reviews, two short surveys, one editorial, three book chapters, and two conference papers (Figure 1). Most included studies (117 papers) were published between 2021 and 2025, reflecting a recent surge in research interest in the connection between air pollution and gut microbiota. A total of 37 papers were published between 2016 and 2020, while only five papers were published between 2010 and 2015, highlighting that this is an emerging area of research. After abstract and full-text review, 158 studies were retained for inclusion in the final analysis.

Trends in Publication and Research Focus

**Figure 2:**
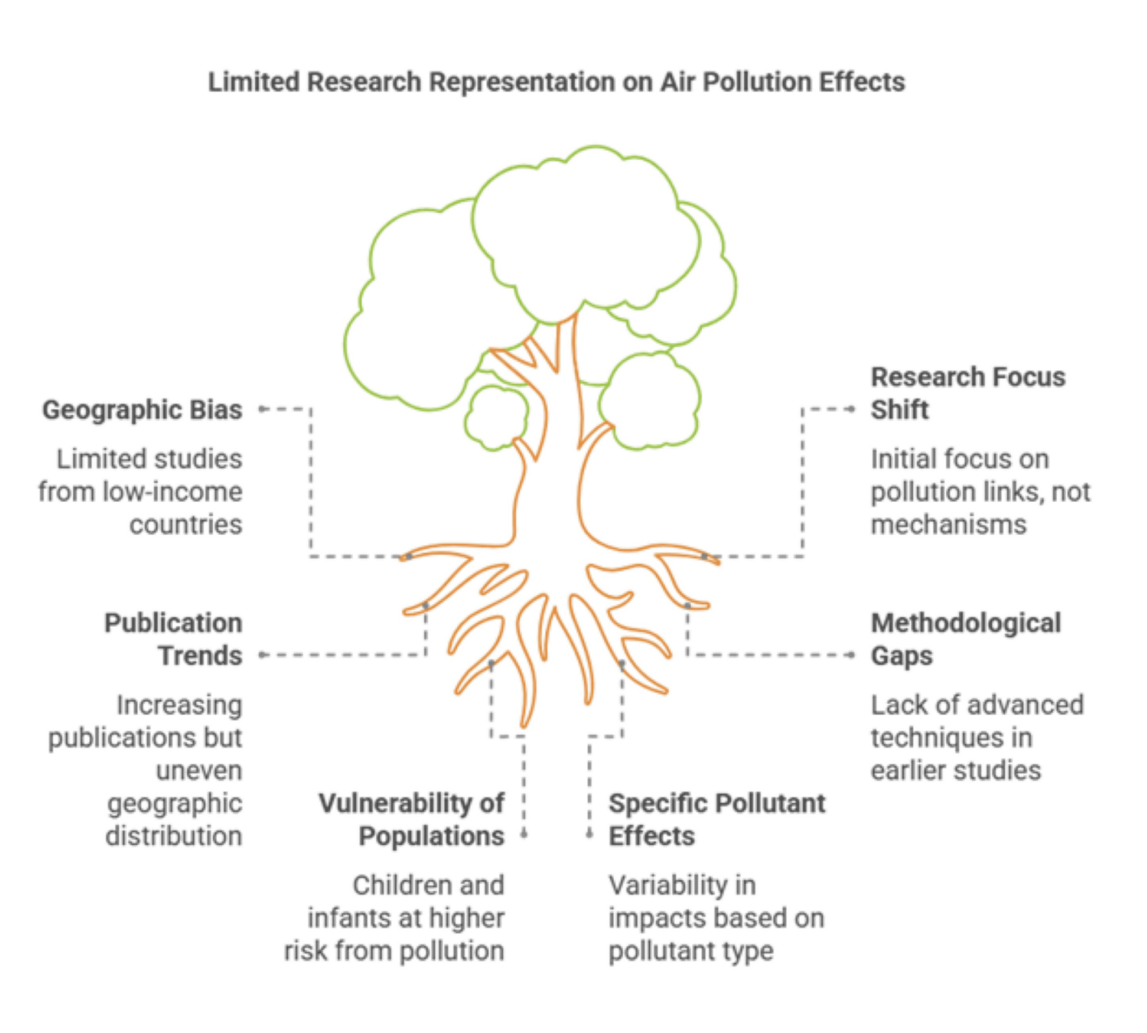
Summary of the main findings. Credit: The figure was created by the first author, Treesa Thomas.

Over time, the increasing number of publications suggests a growing recognition of the role of environmental factors in modulating gut microbiota and influencing human health. Research from 2010 to 2015 primarily focused on identifying early links between air pollution and gut health, with a limited understanding of underlying mechanisms. From 2016 to 2020, the research focus expanded to explore specific pollutants, such as PM2.5, VOCs, and heavy metals, alongside emerging insights into the gut-lung and gut-brain axes. Studies published from 2021 onward demonstrated a more sophisticated understanding of these mechanisms, incorporating high-throughput sequencing, metagenomics, and microbiota-targeted interventions (Figure 2).

Geographic Distribution of Studies

The studies included in this review were geographically diverse, with a significant proportion conducted in China, the United States, India, Japan, and Europe. However, research from low- and middle-income countries (LMICs) remains limited despite higher levels of air pollution exposure in these regions. The predominance of research from East Asia and North America underscores the need for broader geographic representation to enhance the generalizability of findings.

Key Findings on Gut Microbiota Alterations

Across the included studies, exposure to air pollutants was consistently associated with changes in gut microbiota composition and function. Disruption in the balance of major microbial phyla, including Firmicutes, Bacteroidetes, Proteobacteria, Actinobacteria, and Verrucomicrobia, is a frequently reported phenomenon. For example, Wang et al. [12] demonstrated that exposure to ambient PM causes a shift in the Firmicutes/Bacteroidetes ratio, which is closely linked to metabolic and inflammatory disorders. Similarly, Zhou et al. [13] reported that exposure to ammonia reduces gut microbial diversity and triggers TLR4-mediated inflammation, highlighting the immune-modulating effects of air pollution.

Studies also highlighted pollutant-specific effects. For example, Li et al. [14] found that exposure to vehicle exhaust impairs locomotion and increases intestinal permeability in *Drosophila*, mediated by the disruption of gut microbiota and the activation of immune pathways. Zhao et al. [15] linked PM2.5 exposure to insulin resistance and metabolic dysfunction through altered sphingolipid metabolism and gut microbial imbalance. Furthermore, Liu et al. [16] demonstrated that exposure to oil mist particulate matter (OMPM) reduces SCFA production, increases visceral fat accumulation, and activates the TLR4-NF-κB inflammatory pathway.

Gut-Lung Axis and Respiratory Health

The gut-lung axis emerged as a key pathway linking air pollution-induced gut dysbiosis to respiratory health outcomes. Several studies highlighted the impact of altered gut microbiota on pulmonary inflammation, chronic obstructive pulmonary disease (COPD), and asthma. Gupta et al. [17] found that *L. plantarum* alleviates PM-induced pulmonary inflammation by restoring gut microbial balance and reducing oxidative stress. Simon et al. [9] demonstrated that *B. megaterium *(Renuspore®) detoxifies heavy metals and produces beneficial SCFAs, which improve gut-lung communication and reduce respiratory inflammation. Cheng et al. [18] further demonstrated that exposure to PM2.5 leads to epithelial hyperproliferation and the release of inflammatory cytokines through the gut-lung axis.

Gut-Brain Axis and Neurological Health

The gut-brain axis influences the cognitive and neurological effects of air pollution. Yuan and colleagues [19] reported that exposure to indoor air pollution in rural China was associated with cognitive decline, with gut microbial changes (increased *Escherichia*-*Shigella* and reduced *Faecalibacterium*) and elevated oxidative stress playing a key role. Zhou et al. [20] demonstrated that alterations in the gut microbiota mediate the effects of air pollution on brain structural changes and neurodegenerative diseases, such as multiple sclerosis and Alzheimer's disease. Sun et al. [21] further linked oxytocin receptor (OXTR) gene polymorphisms to increased susceptibility to air pollution-induced gut dysbiosis and cognitive disturbances in children.

Metabolic and Cardiovascular Implications

Dysbiosis driven by air pollution was also consistently linked to metabolic and cardiovascular dysfunction. Zhao et al. [15] reported that exposure to PM2.5 increases insulin resistance by altering the gut microbial balance and lipid metabolism. Liu et al. [16] found that PM exposure reduces the number of SCFA-producing bacteria, increases inflammatory markers, and promotes the accumulation of visceral fat. Yaohan et al. [22] demonstrated that specific bacterial species, such as *Bifidobacterium*, are negatively associated with body fat percentage, highlighting the role of gut microbiota in regulating metabolic health.

Protective and Therapeutic Strategies

Several studies proposed therapeutic approaches to lessen the effects of air pollution on gut health. Probiotics and synbiotics have emerged as promising interventions. Gupta et al. [17] found that *L. plantarum* restores gut microbial balance and improves lung health in individuals exposed to PM. Simon et al. [9] demonstrated that *B. megaterium* (Renuspore®) detoxifies pollutants and promotes beneficial SCFA production, reducing inflammation and oxidative stress. Dietary interventions, including fish-derived proteins and fruit-derived biomolecules, also showed potential for reducing oxidative stress and restoring gut homeostasis [8].

Early-Life and Long-Term Exposure Effects

Several studies highlighted the vulnerability of infants and children to air pollution-induced gut dysbiosis. Bailey et al. [23] reported that six-month postnatal exposure to PM10 and PM2.5 alters the infant gut microbiota composition, increasing inflammatory taxa such as *Enterococcus* and *Clostridium* while reducing beneficial bacteria, including *Alistipes*. Lin et al. found that air pollution, fine particles, can cause insulin resistance and adipose inflammation, which can lead to energy reallocation and aberrant growth in infants [24]. Similarly, Sun et al. [21] showed that early-life PM exposure reduces SCFA production, contributing to increased cortisol levels and oxidative stress. Long-term exposure was associated with metabolic and neurological consequences, including cognitive decline, obesity, and insulin resistance (Table 2).

**Table 2:** Characteristics and key findings of the reviewed studies PM: particulate matter; GMMs: genetically modified microorganisms; PM2.5: fine particulate matter; PM10: coarse particulate matter; PM1: ultrafine particulate matter; SCFA: short-chain fatty acids; TLR4: toll-like receptor 4; COPD: chronic obstructive pulmonary disease; ALD: alcoholic liver disease; NAFLD: non-alcoholic fatty liver disease; AECOPD: acute exacerbation of chronic obstructive pulmonary disease; CKD: chronic kidney disease; UFP: ultrafine particles; OXTR: oxytocin receptor; GPR43: g-protein-coupled receptor 43; NF-κB: nuclear factor kappa b; GDM: gestational diabetes mellitus; CSLGI: chronic systemic low-grade inflammation; OP: organophosphorus pesticides; DEP: diesel exhaust particles; Nrf2: nuclear factor erythroid 2-related factor 2; DUOX: dual oxidase; Imd: immune deficiency pathway; Ntcp: sodium-taurocholate cotransporting polypeptide; PTSD: post-traumatic stress disorder; SHOW: Survey of the Health of Wisconsin; ASD: autism spectrum disorder; GI: gastrointestinal; DASH: Dietary Approaches to Stop Hypertension; MIND: Mediterranean-DASH Diet Intervention for Neurodegenerative Delay; AD: Alzheimer’s disease; IBD: inflammatory bowel disease; RA: rheumatoid arthritis; AE: atopic eczema; NASH: non-alcoholic steatohepatitis; PAH: polycyclic aromatic hydrocarbon; PCB: polychlorinated biphenyl; NLRP3: nod-like receptor pyrin domain-containing protein 3; DAMPs: damage-associated molecular patterns; PPAR: peroxisome proliferator-activated receptor; BIOMAP: Biomarkers in Atopic Dermatitis and Psoriasis; PES MNP: polystyrene micro- and nanoplastics; ADHD: attention deficit hyperactivity disorder; PAMPs: pathogen-associated molecular patterns

Author	Year	Country	Methods	Main findings
Kozyrskyj et al. [25]	2011	Canada	Review	Prenatal and early-life environmental exposures, including stress, air pollution, and diet, influence the development of asthma and allergic diseases by shaping the infant gut microbiota, with protective factors like Vitamin D intake and a Mediterranean diet. In contrast, the role of early-life fever and infections remains debated.
Salim et al. [3]	2013	Canada	Article	PM in urban air may be a factor in the increased prevalence of inflammatory bowel disease by altering the gut microbiome, immune function, and intestinal barrier, suggesting its role as a potential risk factor for IBD.
Haahtela et al. [26]	2013	Finland	Article	Biodiversity loss and air pollution are interlinked factors that negatively affect gut microbiota, contributing to inflammatory diseases and highlighting the need for global strategies to preserve biodiversity, reduce pollution, and promote microbial resilience to improve health.
Wiernsperger [27]	2014	France	Article	Hepatic steatosis, closely linked to cardiometabolic disorders and diabetes, arises from dietary, intestinal, hepatic, and epigenetic factors, with gut microbiota playing a key role, highlighting the need for personalized, microbiome-based therapies and long-term clinical trials to refine treatment strategies.
Martin et al. [28]	2015	UK	Article	Factors contributing to relapses in Crohn's disease and ulcerative colitis include medications, smoking, stress, diet, antibiotic use, air pollution, and changes in gut microbiota, highlighting the need for further research to improve management strategies.
Abegunde et al. [29]	2016	USA	Review	The development of IBD results from interactions between genetics, immune dysregulation, gut microbiota imbalances, and environmental exposures, with rising incidence highlighting the need for research into modifiable risk factors, comprehensive models, and integrative approaches to enhance prevention, diagnosis, and treatment.
Adar et al. [30]	2016	USA	Review	The respiratory microbiome plays a crucial role in chronic lung diseases by metabolizing pollutants, modulating inflammation, and influencing susceptibility to air-borne toxins. This highlights the need for further research to improve public health strategies and interventions for vulnerable populations.
Rosenfeld [31]	2017	USA	Review	Environmental chemical exposures, including endocrine disruptors, heavy metals, nanoparticles, and air pollution, can alter gut microbiota composition, leading to dysbiosis and contributing to diabetes, obesity, and GI, immunological, and neurobehavioral disorders through bacterial metabolites, hormone-like substances, and epigenetic changes.
Chotirmall et al. [32]	2017	Singapore	Review	This review examines the impact of microbiome changes influenced by environmental factors on respiratory diseases such as COPD, asthma, cystic fibrosis, and bronchiectasis. It highlights the role of gut microbiomes, diet, antibiotics, and challenges in establishing cause-and-effect relationships to improve treatment strategies, particularly in the Asia-Pacific region.
Alderete et al. [33]	2018	USA	Article	Traffic-related air pollution exposure increases alterations in gut microbiota composition among overweight and obese adolescents, resulting in a decrease in *Bacteroidaceae* and an increase in *Coriobacteriaceae*. These changes correlate with elevated fasting glucose levels and may contribute to metabolic dysfunction, raising the risk of type 2 diabetes.
Mutlu et al. [34]	2018	USA	Article	In a murine model, exposure to PM caused changes in gut microbiota diversity, with alterations in bacterial genera that may contribute to GI inflammation and offer insight into PM-induced GI health issues.
Vallès and Francino [35]	2018	Barbados	Review	Early-life changes in the microbiome, influenced by factors like reduced microbial exposure, antibiotic use, and poor diet, can impact immune, metabolic, and neurological development, potentially leading to diseases such as atopic conditions, autism, and obesity.
Ananthakrishnan et al. [36]	2018	USA	Review	This review highlights the complex role of environmental factors, including dysbiosis, early-life events, air pollution, and high-altitude hypoxia, in the initiation and progression of IBD. It emphasizes the need for multifaceted therapies and systems biology to understand and address the diverse environmental influences on its pathogenesis.
Saeed et al. [37]	2018	China	Review	Mojave yucca (*Yucca schidigera*) offers multiple health benefits. It can reduce ammonia emissions and fecal odors in poultry through gut microbiota modifications, providing a cost-effective, natural alternative to expensive pollution control methods.
Kim and Kim [38]	2019	Korea	Article	Atopic dermatitis is influenced by skin barrier defects, immune dysregulation, and microbial dysbiosis, with early-life microbiota changes playing a critical role in immune development, highlighting the potential of microbiome-targeted therapies that require further validation.
Sbihi et al. [39]	2019	Canada	Review	Infant gut microbiome dysbiosis, influenced by individual factors like delivery mode and antibiotics as well as broader environmental exposures such as air pollution and the built environment, disrupts immune regulation and increases the risk of asthma and allergic diseases, highlighting the need for an exposome approach to understand these complex interactions.
Liu et al. [40]	2019	China	Article	Exposure to PM2.5 and PM1 increases the risk of impaired fasting glucose and type 2 diabetes by negatively affecting gut microbiota diversity, with specific bacterial taxa acting as mediators.
Curciarello et al. [41]	2019	Argentina	Review	Exposure to PM2.5 and PM1 increases the risk of impaired fasting glucose and type 2 diabetes by decreasing gut microbiota diversity, with specific bacterial taxa, such as Firmicutes, Proteobacteria, and Verrucomicrobia, mediating this association.
Ristori et al. [42]	2019	Italy	Review	ASD is associated with genetic and environmental factors, with GI issues and altered gut microbiota being common. Dietary interventions that target gut health and SCFAs show potential for improving ASD-related symptoms.
Dempsey et al. [43]	2019	USA	Article	The gut microbiome regulates CNS signaling through mechanisms like neurotoxicant biotransformation, microbial metabolite production, and gut-brain communication, with environmental stressors potentially disrupting these processes and contributing to neurological disorders.
Li et al. [44]	2019	China	Article	Exposure to diesel exhaust particles causes colon epithelial injury, inflammation, and transient changes in gut microbiota, with probiotics offering protection against DEP-induced colonic damage by modulating microbiota and nitrogen metabolism.
Roslund et al. [45]	2019	Finland	Article	Exposure to PAHs, particularly chrysene, in daycare children was linked to altered gut and skin microbiota, including changes in *Mycobacterium*, as well as disruptions in adipocytokine and peroxisome proliferator-activated receptor signaling, suggesting that conventional risk assessments may underestimate health risks due to microbiota-related endocrine disruptions.
Fox et al. [46]	2019	USA	Review	Lifestyle changes, such as agriculture, industrialization, and globalization, have reduced microbial diversity and increased microbial virulence. These changes could potentially heighten AD risk through mechanisms like immune dysfunction, chronic inflammation, and disruption of epithelial barriers. The impact of microbial dysbiosis could be potentially influenced by APOE genotypes.
Turkalj [47]	2019	Croatia	Article	The development of asthma and allergic diseases may be influenced by factors such as individual susceptibility, allergen exposure, infections, and environmental pollutants, with disturbances in the gut and respiratory microbiota playing a crucial role, suggesting that modulating the microbiota, especially in early life, could be a potential strategy for prevention and treatment.
Frati et al. [48]	2019	Italy	Review	The review emphasizes the role of the gut-lung axis, highlighting the importance of early-life exposure to diverse microbiota in preventing asthma, and suggests that gut dysbiosis, along with factors like tobacco smoke and genetics, may contribute to asthma and chronic respiratory inflammation.
Liu et al. [49]	2020	China	Article	Prenatal PM2.5 exposure reduces fetal body weight and length, alters maternal gut microbiota diversity and composition by increasing Proteobacteria and decreasing Acidobacteria, disrupts SCFA levels by elevating isobutyric acid and lowering butyric acid, and may contribute to adverse pregnancy outcomes through gut microbiota-mediated mechanisms.
Fitch et al. [50]	2020	USA	Article	Wood-smoke and mixed vehicle exhaust exposure compromise intestinal barrier integrity, increase inflammation, and alter gut microbiota composition in atherosclerotic mice, potentially accelerating cardiovascular disease progression.
Wan-jun et al. [51]	2020	China	Article	Exposure to wood smoke and mixed vehicle exhaust disrupts gut health in atherosclerotic mice by compromising intestinal barrier integrity, increasing inflammatory markers, and altering gut microbiota composition, potentially accelerating cardiovascular disease progression.
Feng et al. [52]	2020	China	Review	Air pollution, particularly PM, negatively affects the GI system by disrupting lipid metabolism, altering the gut microbiome, and contributing to diseases like appendicitis, inflammatory bowel disease, and colorectal cancer. Thus, the need to consider the GI system in air pollution health studies is emphasized.
Liu et al. [53]	2020	China	Article	Maternal exposure to PM2.5 harms offspring gut health by increasing oxidative stress, inflammation, and altering gut microbiota, particularly Bacteroides, while quercetin supplementation improves antioxidant activity, reduces inflammation, and restores gut barrier function, suggesting it may protect against PM2.5's adverse effects on offspring gut health.
Huang et al. [54]	2020	China	Review	Environmental factors like high-fat diets, smoking, alcohol, and pollution contribute to dysbiosis, which is linked to non-communicable diseases such as cancer and diabetes, with person-to-person transmission of dysbiotic microbiota, including Helicobacter pylori, potentially playing a role in disease spread.
Wang et al. [55]	2020	USA	Article	Exposure to PCBs in a simulated school environment led to modest cognitive impairments, increased oxidative stress in the liver and lungs, changes in hematopoietic stem cell maturation, altered telomerase activity, and gut microbiota disruptions, with tissue PCB levels matching the target school air profile.
Donovan et al. [56]	2020	Australia	Review	The NLRP3 inflammasome, activated by PAMPs and DAMPs, plays a crucial role in promoting inflammation in the gut and lungs, with microbiome crosstalk through the gut-lung axis influencing disease processes, and the development of the NLRP3 inhibitor MCC950 advancing research into treatment strategies.
Liberti et al. [57]	2020	Italy	Article	Exposure to resuspended pollutants, including PAHs and heavy metals, in Ciona robusta induces oxidative stress, mucus overproduction, and crypt lumen occlusion, highlighting the increased disease susceptibility of marine invertebrates to chronic contamination and acute disturbances, potentially worsened by climate change.
Leyva-López et al. [58]	2020	Mexico	Review	Agro-industrial waste, rich in bioactive compounds like phenolics, terpenes, and β-glucans, can be repurposed as food additives to improve the health, growth, and disease resistance of aquatic organisms, offering environmental and sustainability benefits in aquaculture.
Dujardin et al. [59]	2020	USA	Review	Air pollution exposure impacts the gut microbiome in both animals and humans, with animal studies demonstrating changes in β-diversity while human studies show no clear patterns. This discrepancy highlights the need for standardized research methods to gain a better understanding of these effects and their underlying mechanisms.
Narla and Silverberg [60]	2020	USA	Review	Environmental factors such as prenatal exposures, early-life influences, climate factors, and household elements play a significant role in the development of atopic dermatitis, with complex gene-environment interactions making it challenging to identify definitive risk factors, highlighting the need for comprehensive preventive and therapeutic approaches.
DeKruyff et al. [61]	2020	California	Conference	The Keystone Symposium highlighted the role of epithelial damage, influenced by modern environmental factors like altered microbiomes, air pollution, food allergens, and chemicals, in the development of allergic diseases, and discussed strategies such as precision medicine, oral desensitization, gut microbiome modifications, and behavioral changes for prevention and treatment.
Bailey et al. [23]	2020	USA	Review	The review highlights how exposure to air pollutants, such as PM, ozone, and nitrogen oxides, alters the gut microbiota, potentially increasing the risk of obesity and type 2 diabetes through inflammatory pathways, and calls for further research to explore these complex interactions.
Fouladi et al. [62]	2020	USA	Articles	Exposure to air pollutants, particularly ozone and nitrogen dioxide, is significantly associated with changes in gut microbiome composition and function, including decreased diversity, increased *Bacteroides caecimuris*, and alterations in key biosynthesis pathways.
Zheng et al. [63]	2020	China	Article	Short-term air pollution exposure caused significant shifts in the gut microbiome of asthmatic children, decreasing beneficial bacteria and increasing harmful ones, which may trigger asthma attacks by altering immune-related gut bacteria.
Di Tommaso et al. [64]	2021	Italy	Review	The intestinal mucosa maintains a selective barrier for nutrient absorption and protection, with gut microbiota playing a crucial role, while disruptions from pathogens, xenobiotics, food, and genetic and immune factors contribute to gut dysbiosis and inflammation, linking it to metabolic and autoimmune diseases.
van den Brule et al. [65]	2021	Belgium	Article	Diesel exhaust particle exposure led to dose-dependent gut microbiota alterations, including reduced α-diversity and changes in bacterial composition, but did not significantly impact cardiovascular or metabolic health, suggesting the need for further research on its long-term effects.
Cabrera et al. [66]	2021	Spain	Review	Addressing modifiable risk factors like obesity, hypertension, and diabetes could prevent or delay up to 40% of dementia cases, while gut dysbiosis contributes to metabolic dysfunction and inflammation, suggesting that microbiota-targeted interventions may offer a promising strategy for dementia prevention.
Vignal et al. [67]	2021	France	Review	Air pollution, through both gaseous and particulate pollutants, disrupts gut microbiota, impairs oxidative balance, triggers inflammation, and compromises gut integrity, highlighting the need for further research to understand its role in GI diseases.
Yang et al. [68]	2021	China	Article	Second-hand smoke exposure disrupts hepatic metabolism, increases liver cholesterol accumulation, impairs bile acid homeostasis, induces insulin resistance, and alters gut microbiota composition, especially under high-fat diet conditions, emphasizing the need for smoking bans to protect metabolic and gut health.
Anwar et al. [69]	2021	Pakistan	Article	The human microbiota, a dynamic and symbiotic ecosystem shaped by diet, medications, and environmental factors, plays a vital role in physiological functions, with its balance being crucial for health, and dysbiosis potentially leading to disease, emphasizing the need for deeper research into its complex interactions.
Wang and McDonald [70]	2021	China	Review	The 2019/2020 bushfires worsened asthma symptoms, while controlled asthma did not increase COVID-19 severity, highlighting the need for personalized medicine, cautious oral corticosteroid use, effective biologic therapies, and further research into lung, gut, and skin microbiome dysregulation in asthma pathogenesis.
Nowak et al. [71]	2021	Poland	Review	Honeybee health is influenced by their natural microbiota, particularly lactic acid bacteria, and probiotics are proposed as a promising alternative to antibiotics and biocides for improving their well-being and preserving populations.
Cui et al. [72]	2021	China	Review	The rising incidence of IBD in China is closely associated with industrialization, urbanization, and economic growth, with factors such as Westernized diets, obesity, and work-related stress contributing to gut microbiota disruption and highlighting the need for further research on environmental and genetic interactions in IBD.
Fournier et al. [73]	2021	France	Article	Human exposure to microplastics, primarily through the GI tract, disrupts gut homeostasis by affecting microbiota, mucus production, and epithelial integrity, while also carrying contaminants like heavy metals and pathogens, highlighting the need for further research and advanced models to understand its long-term effects on intestinal health.
Lu et al. [74]	2021	China	Review	PM exposure increases intestinal permeability, impairs the immune barrier, alters gut microbiota composition, and triggers inflammatory responses, highlighting the need for further research to understand its impact on human health through the intestinal tract.
Earp et al. [75]	2021	UK	Review	Parental atopy, being born in autumn or winter, and environmental factors like probiotics, maternal antibiotic use, and air pollution are associated with AE risk, though evidence on gene-environment interactions and other risk factors, such as yoghurt consumption and feeding practices, remains inconsistent and limited.
Paul et al. [76]	2021	Bangladesh	Review	RA pathogenesis is influenced by genetic, epigenetic, and environmental factors, including smoking, air pollution, diet, and GI microbiota imbalancbacillus probiotics, showing potential in managing RA, though their exact mechanism of action remains unclear.
Sheina et al. [77]	2021	Russia	Article	Inhalation exposure to biotechnological microorganism strains, including yeasts, molds, and gram-negative bacteria, caused significant shifts in gut microbiota in rats, decreasing *Escherichia coli* and increasing gram-positive bacteria, emphasizing the need for hygienic standards to mitigate occupational and residential risks.
Stefanovic et al. [78]	2021	Ireland	Review	Environmental exposures, including climate change, migration, air pollution, and diet, significantly impact the pathogenesis of atopic dermatitis by affecting the skin barrier, immune response, and microbiota, with interactions between these factors and genetic susceptibility influencing disease development.
Wang and Dykes [79]	2021	Australia	Article	Emerging evidence suggests that modulating the gut microbiota through lifestyle and dietary interventions may offer a promising avenue for preventing or mitigating AD by addressing the gut-brain axis, though further research is needed to clarify causal links and personalize treatment strategies.
Juanola et al. [80]	2021	Switzerland	Review	The growing prevalence of NAFLD is driven by obesity, metabolic diseases, and environmental factors, with its progression to NASH influenced by insulin resistance, diet, gut microbiota, and genetic and epigenetic factors, particularly in genetically predisposed individuals.
Alenius et al. [81]	2021	Swedan	Review	The BIOMAP project aims to explore the causes and mechanisms of atopic dermatitis and psoriasis, focusing on clinical heterogeneity, biomarkers, and the role of microbial exposures and microbiota in immune polarization and disease pathogenesis, through a collaborative approach involving industry and academic partners.
Li et al. [82]	2021	China	Article	Fluoride exposure in ducks leads to impaired intestinal structure, a reduction in goblet cells, decreased alpha diversity in gut microbiota, and the absence of specific bacterial phyla and genera, highlighting significant damage to both intestinal health and microbial composition.
Vari et al. [83]	2021	Finland	Article	High coverage of broad-leaved and mixed forests near homes was associated with lower PAH levels in ambient air and increased gut functional orthologues for the PPAR pathway, suggesting that forests may reduce PAH exposure and mitigate pollution-induced disruptions in the gut microbiota, potentially lowering endocrine disruption risks.
Zhang et al. [84]	2021	China	Article	Gestational PM2.5 exposure is linked to reduced alpha diversity and altered beta diversity in the neonatal meconium microbiome, with significant shifts in bacterial composition and functional pathways related to metabolism and transport.
Pulliero et al. [85]	2021	Italy	Review	Environmental pollutants disrupt microbiota diversity and the lung-gut axis, influencing inflammatory diseases and cancer progression through microbiota-derived metabolites and epigenetic modifications, while metagenomics and microbiota monitoring offer potential strategies for disease prevention and intervention.
Zhou et al. [13]	2022	China	Article	Ammonia exposure induces respiratory tract injuries, particularly tracheal damage, by disrupting gut and tracheal microbiota, with TLR4-mediated inflammation playing a key role, suggesting that gut microbiota modulation could be a potential therapeutic target for mitigating ammonia-induced injuries.
Gupta et al. [5]	2022	India	Review	Air pollution-driven dysbiosis extends beyond the gut microbiota, disrupting microbial communities in the oral, nasal, respiratory, skin, and thyroid regions, potentially leading to multi-organ health risks.
Santa et al. [86]	2022	Japan	Review	Grape phytochemicals and vitamin D help mitigate lung diseases like pneumonia, asthma, and COPD by reducing inflammation, boosting immunity, supporting gut microbiota balance, and promoting healthy dietary and lifestyle choices, which are crucial for prevention and improving outcomes in chronic lung conditions.
Li et al. [14]	2022	China	Article	Vehicle exhaust exposure shortens lifespan, impairs locomotion, disrupts gut microbiota, increases intestinal permeability, and cell proliferation in *Drosophila*, with Imd and DUOX activation playing a key role.
Basarkar et al. [87]	2022	India	Review	Transformative potential of GMMs in healthcare, particularly in obesity, cancer, diabetes, HIV, malaria, and inflammatory bowel disease, emphasizing their precision, reduced side effects, and expansion into other fields, while stressing the need for regulatory oversight and ethical integration.
Sommer et al. [88]	2022	United States	Article	A causal inference framework reveals that environmental factors, such as smoking prevention, can influence gut microbiome composition, notably affecting microbial taxa like Christensenellaceae and Ruminococcaceae.
Li et al. [89]	2022	China	Article	Hexavalent chromium (Cr VI) exposure significantly reduces gut microbial alpha-diversity in chickens, alters taxonomic composition by decreasing two phyla and 47 genera while increasing three phyla and 17 genera, and eliminates nine genera, including *Coprobacter* and *Ruminococcus_1*, highlighting its disruptive impact and potential for mitigation through gut microbiota modulation.
Bailey et al. [90]	2022	USA	Article	Six-month postnatal exposure to ambient air pollution (PM10, PM2.5, and NO2) alters infant gut microbiota composition by increasing genera such as *Dialister, Dorea, Acinetobacter, Campylobacter, Actinomyces, Enterococcus, Clostridium, and Eubacterium* while decreasing *Alistipes*, with many affected taxa linked to systemic inflammation, highlighting potential implications for infant health and development.
Habotta et al. [7]	2022	Egypt	Review	Non-edible fruit by-products from industrial processes contain bioactive compounds with antioxidant, immune-boosting, and gut microbiota-modulating properties, offering sustainable value as feed additives in aquaculture while reducing environmental impact and supporting healthier farming practices.
Dutta et al. [91]	2022	USA	Article	Chronic exposure to traffic-related air pollution alters gut microbiota and bile acid metabolism in a sex- and genotype-specific manner, reducing microbial diversity in male TgF344 AD rats, shifting inflammation-associated bacteria in females, increasing primary and secondary bile acids, and downregulating Ntcp in males, potentially contributing to AD risk.
Mbareche et al. [92]	2022	Canada	Article	Wastewater treatment plants harbor dynamic microbial communities, generating bioaerosols with site-specific bacterial diversity, persistent gut-associated flora, and pathogenic taxa that peak during degritting and degreasing, posing occupational risks and underscoring the need for enhanced monitoring and protective measures for workers.
Sun et al. [21]	2022	China.	Article	The OXTR gene polymorphism (rs53576) influences the relationship between air pollution (PM2.5) and gut microbiota in children, with high pollution exposure increasing cortisol, oxidative stress markers, and decreasing beneficial SCFAs, while children with GA/GG genotypes were more susceptible to microbiota disturbances than AA carriers, highlighting the need for targeted interventions to mitigate pollution’s impact on children’s health and neurodevelopment.
Mundula et al. [93]	2022	Italy	Review	Modern lifestyle factors such as poor diet, stress, smoking, alcohol abuse, inactivity, and pollution disrupt gut microbiota, exacerbating CSLGI and contributing to inflammation-linked diseases like long-COVID, highlighting the need for lifestyle modifications and targeted interventions to reduce chronic inflammation and improve health outcomes.
Phillippi et al. [94]	2022	USA	Article	Inhaled DEP exposure caused shifts in the gut microbiome, reduced Actinobacteria, and expanded Verrucomicrobia and Proteobacteria, while elevating inflammatory cytokines and cardiovascular disease biomarkers, with probiotic intervention mitigating these effects.
Keulers et al. [95]	2022	Netherlands	Review	Air pollution significantly impacts GI and respiratory health through the gut-lung axis, with microbial metabolites and immune mediators influencing disease progression, while synbiotics show potential in reducing inflammation and oxidative stress, though further clinical research is needed to confirm their efficacy.
Smith and Melrose [96]	2022	Australia	Review	Prebiotic xylans support gut health by fostering beneficial microbiota, preventing pathogen colonization, reducing antibiotic dependence, lowering methane emissions in livestock, and offering therapeutic potential for conditions like osteoarthritis, highlighting the need for further research on their broader health applications.
Malecki et al. [97]	2022	USA	Article	The SHOW provides extensive data on social determinants, environmental exposures, and health outcomes from over 6,800 participants, supporting research on cardio-metabolic disparities, microbiome health, antibiotic resistance, air pollution, and aging while serving as a crucial resource for longitudinal and translational public health studies.
Pambianchi et al. [98]	2022	USA	Conference Paper	Airborne particulate matter not only contributes to respiratory diseases but also causes GI damage through direct exposure and systemic inflammation, with the lung-gut axis playing a key role, as seen during the COVID-19 pandemic, underscoring the need to understand its mechanisms and health impacts.
Filardo et al. [99]	2022	Italy	Review	Air pollution alters gut microbiota composition, increasing the risk of chronic diseases like impaired fasting glucose, adverse pregnancy outcomes, and asthma, highlighting the need for larger cohort studies and targeted interventions to mitigate these health risks.
Gan et al. [100]	2022	China	Article	Air pollution exposure during pregnancy increases the risk of adverse pregnancy outcomes (1.07 to 1.36-fold) by altering gut microbiota composition, with pollutants like PM2.5, PM10, O_3_, NO_2_, and SO_2_ influencing bacteria (*Micrococcaceae, Rothia, Parabacteroides*), highlighting the need for mitigation strategies to protect maternal and fetal health.
Wang et al. [101]	2022	China	Article	Temperature influences zebrafish radiosensitivity by altering gut microbiota configurations, with antibiotic treatment reducing radiation effects and gene expression differences, suggesting that maintaining stable gut microbiota could protect aquatic organisms from radiation and climate change-induced temperature changes.
Mousavi et al. [102]	2022	Review	Iran	Environmental toxicants, air pollutants, and endocrine disruptors disrupt the human microbiome across multiple body systems by increasing harmful bacteria like Streptococcus and Veillonellales, altering microbial diversity, and potentially promoting pathogen colonization and metabolic dysfunction.
Zhao et al. [15]	2022	China	Article	PM2.5 exposure in the elderly contributes to insulin resistance through systemic inflammation, sphingolipid metabolism alterations, and gut microbiota modulation, emphasizing air pollution as a risk factor for metabolic diseases like type 2 diabetes and the need for mitigation strategies and microbiome-targeted interventions.
Alharbi et al. [103]	2022	Saudi Arabia	Book Chapter	Asthma development is influenced by factors like genetics, infections, and environmental exposures, with gut dysbiosis and microbiome disruptions playing a crucial role in immune regulation and asthma severity, highlighting the need for further research on the microbiota's impact on asthma risk.
Dai et al. [104]	2022	China	Article	PM2.5 exposure in male Balb/C mice led to initial weight loss followed by recovery, accompanied by gut microbiota dysbiosis, increased *Lactobacillus* and *Clostridium*, and disruptions in arachidonic acid, prostaglandin pathways, and PPAR signaling, potentially contributing to liver injury, ileum inflammation, and weight regulation issues.
Fadlyana et al. [105]	2022	Indonesia	Review	Air pollution adversely impacts children's health by influencing immunity, neurodevelopment, and metabolism through immune and allergic pathways, with gut microbiota alterations as a potential mediator, necessitating further multidisciplinary research and public health interventions.
Simon et al. [9]	2023	United States	Article	Renuspore® (*Bacillus megaterium* MIT411) shows promise as a probiotic for mitigating the impact of environmental pollution on gut microbiota by detoxifying heavy metals, degrading pollutants like nitrite and ammonia, and producing beneficial metabolites such as SCFAs, which may help counteract gut dysbiosis linked to air pollution exposure.
Boroujeni et al. [106]	2023	Iran	Review	Air pollution contributes to functional GI disorders by promoting gut microbiota dysbiosis, inflammation, and motility disruptions, while dietary interventions like probiotics and prebiotics may help mitigate these effects alongside pollution reduction policies.
Wang et al. [107]	2023	China	Article	PM2.5 exposure induces microbiome dysbiosis in both the lung and gut, with more severe disruptions in Nrf2 knockout mice compared to wild-type mice. This highlights Nrf2's protective role in maintaining microbiome homeostasis and mitigating organ-specific toxicity from environmental pollutants.
Zhao et al. [108]	2023	China	Article	Pre- and persistent exposure to PM2.5 in aged rats impaired lung function, worsened asthma symptoms, and altered cytokines, immunoglobulins, gut microbiota, and plasma metabolites. Elevated PAHs like naphthalene suggested a mechanistic link between air pollution, metabolic disorders, and asthma exacerbation through microbiota-mediated immune responses.
Liu et al. [109]	2023	China	Review	The gut-lung-kidney axis in immune-related CKD involves microbiota dysbiosis, intestinal barrier dysfunction, and immune regulation, with CKD exacerbating respiratory conditions and environmental factors like PM influencing immune responses, while therapeutic strategies such as prebiotics, probiotics, and *Rheum officinale *may restore gut ecology, reduce oxidative stress, and improve immune balance, highlighting the importance of gut health in managing CKD progression.
Chelu et al. [110]	2023	Romania	Article	Early identification and management of risk factors for GDM, including obesity, family history, hypertension, stress, air pollution, and gut microbiota alterations, along with interventions like weight management, lifestyle changes, and microbiota analysis, can prevent long-term health consequences for both mothers and children. This highlights the need for further research to refine prevention strategies.
Mazumder et al. [111]	2023	USA	Article	Co-exposure to carbon black and ozone in mice induced lung microbial dysbiosis with reduced diversity and increased *Clostridiaceae* and *Prevotellaceae*. The gut microbiome showed elevated SCFA-producing bacteria, altered metabolic receptor expression, and heightened oxidative stress, suggesting a compensatory homeostatic shift.
Goveas et al. [112]	2023	India	Article	Microplastics are pervasive in ecosystems and harmful to organisms. While biological remediation, especially via insect gut microbiota, shows potential for sustainable degradation, further research is needed to enhance its efficiency and understand the underlying mechanisms.
Qiu et al. [113]	2023	China	Article	PM exposure in toddlers reduces gut microbiota α-diversity, with higher exposure to PM1, PM2.5, and PM10 decreasing *Enterococcus* abundance and increasing *Ruminococcaceae* and *Lachnospiraceae*. Lagged effects indicate that the timing of exposure influences microbiota composition, emphasizing the need for further research on the long-term impact of PM on gut health in young children.
Li et al. [114]	2023	China	Article	Increased PM2.5 exposure in AECOPD patients was associated with decreased gut microbiome diversity, specific microorganisms linked to glycolysis, and disturbances in lipid and fatty acid metabolism. This highlights the potential for biomarkers to guide precision strategies for COPD prevention and management while emphasizing the impact of air pollution on gut health and metabolism.
Sadeghi et al. [115]	2023	Iran	Review	Climate change adversely affects GI health by increasing infections and disorders through environmental stressors, air pollution, and microbiota disruptions. This highlights the need for preventive strategies, policy action, and research into microbiome-based interventions.
Van Pee et al. [116]	2023	Belgium	Review	Ambient particulate air pollution negatively impacts gut microbiome diversity and composition, with specific shifts in taxa like increased Bacteroidetes, Deferribacterota, and Proteobacteria and decreased Verrucomicrobiota. This suggests potential long-term health risks and highlights the need for further research into the underlying mechanisms.
Barouki et al. [117]	2023	France	Review	Exposomics, which incorporates environmental factors like air pollution, chemical contaminants, radiation, microbial metabolites, and the gut-liver axis, is advancing our understanding of liver diseases and offering new biomarkers and therapeutic targets to improve prevention and treatment strategies.
Aribi et al. [118]	2023	Algeria	Editorial	Vitamin D plays a crucial role as an immunomodulator, with studies highlighting its timing-dependent effects on immune responses, differential impacts of vitamin D2 and D3 on the blood transcriptome, regulation of immune and skeletal muscle responses in athletes, and potential in preventing deep vein thrombosis. It also benefits autoimmune diseases like type 1 diabetes and reduces inflammation in type 2 diabetes.
Shen et al. [119]	2023	China	Review	The intestinal mucosa, regulated by the gut microbiota, maintains a selective barrier for nutrient absorption and protection. Disruptions from pathogens, xenobiotics, food, and genetic and immune factors lead to inflammation, tissue damage, and gut dysbiosis, which is linked to 'leaky gut syndrome' and metabolic and autoimmune diseases.
Korbush et al. [120]	2023	Ukraine	Article	PM from cottonwood combustion and medical masks significantly affects *E. coli* B906 by inhibiting growth, altering biofilm formation, and increasing antibiotic resistance, raising concerns about the impact of PM exposure on gut microbiota and antimicrobial resistance.
Dorofeyev et al. [121]	2023	Ukraine	Article	Higher air pollution levels exacerbate intestinal damage and microbial dysbiosis in ulcerative colitis and irritable bowel syndrome patients. Those in highly polluted regions show more severe mucus alterations, cellular infiltration, decreased *Akkermansia muciniphila*, and increased Proteobacteria, highlighting the detrimental impact of PM2.5 on gut health.
Abdelkawi et al. [122]	2023	United States	Book Chapter	Psychological, environmental, and physical stressors alter gut microbiota composition by reducing diversity, increasing pro-inflammatory bacteria, and disrupting balance through hormone and immune system interactions, contributing to conditions like IBD and other health issues.
Singh et al. [123]	2023	India	Review	Ozone exposure, particularly in urban areas of low-income nations, is linked to neurotoxic effects, neural damage, behavioral changes from prenatal and postnatal exposure, gut microbiota disruption, oxidative stress, and cellular damage. This highlights the need for further research on its impact and biomarkers for assessment.
Cheng et al. [124]	2023	Taiwan	Review	Cigarette smoking and PM exposure contribute to insulin resistance, hyperglycemia, and gut dysbiosis by disrupting the lung-gut axis and reducing SCFA levels, which impairs anti-inflammatory pathways and metabolic regulation, underscoring SCFAs as a potential therapeutic target.
Kim et al. [125]	2023	USA	Book Chapter	Gut microbiome dysbiosis in ASD is associated with neurodevelopmental, behavioral, and GI symptoms. Microbiome and immune-targeted interventions - such as diet, probiotics, antibiotics, and cytokine blockade - offer potential therapeutic benefits across preconception to postnatal stages.
Yao et al. [126]	2023	China	Article	Soluble dietary fiber from *Prunus persica* dregs reduced lead bioaccumulation, enhanced excretion, preserved gut barrier integrity, modulated gut microbiota by increasing *Desulfovibrio* and *Alistipes*, and altered metabolites associated with detoxification, suggesting a protective role against lead-induced toxicity.
Ben-Azu et al. [127]	2023	Canada	Review	Schizophrenia is linked to gut microbiota dysbiosis that influences brain aging and microglial function via the gut-brain-microglia axis, with environmental factors modulating this interaction and psychobiotics emerging as promising adjuncts to antipsychotic therapy.
Jabin et al. [128]	2023	USA	Article	Biomass fuel use and elevated PM2.5 exposure in Bangladeshi households were linked to adverse birth outcomes and increased infant respiratory infections. In contrast, distinct gut and respiratory microbiomes were observed in infants, emphasizing the impact of environmental and socioeconomic factors on early-life health.
Li et al. [129]	2023	China	Article	Long-term exposure to PM2.5 and its constituents, particularly black carbon, reduced gut microbial diversity and altered composition by decreasing Bacteroidetes and increasing Proteobacteria, underscoring the impact of air pollution on gut health and the potential benefits of controlling black carbon emissions.
Yuan et al. [19]	2024	China	Article	Indoor air pollution in rural northwest China is linked to cognitive impairment in 67.5% of elderly residents, particularly females, through increased exposure to PM1, PM2.5, NO2, CO, and O3, reduced oxidative stress biomarkers, decreased gut microbial diversity, lower *Faecalibacterium*, and higher *Escherichia_Shigella* and *Akkermansia* levels. This suggests a role of inflammation, oxidative stress, and gut dysbiosis in cognitive decline.
Gupta et al. [17]	2024	Saudi Arabia	Short Survey	The effects of PM2.5, which cause inflammation and hyperresponsiveness and contribute to respiratory diseases, may be lessened by *Lactiplantibacillus plantarum*'s antioxidant properties, regulation of the gut-lung axis, and production of SCFA.
Yaohan et al. [22]	2024	China	Review	Exposure to ambient PM disrupts gut microbiota homeostasis, particularly affecting the abundance and function of key phyla - Firmicutes, Bacteroidetes, Proteobacteria, Actinobacteria, and Verrucomicrobia - and is associated with the development of both respiratory and digestive system diseases.
Liu et al. [8]	2024	China	Review	Fish by-product proteins, rich in collagen and bioactive compounds, offer promising applications in skincare by improving skin health, reducing oxidative stress, and enhancing gut microbiota, with significant potential across multiple industries.
Nair et al. [130]	2024	India	Review	Mast cells, primarily located around hepatic structures, play a crucial role in liver disease progression by increasing after hepatic injury, influencing conditions like ALD, NAFLD, viral hepatitis, fibrogenesis, and hepatocellular carcinoma, while also contributing to gut-liver axis interactions through their role in GI barrier integrity and gut microbiome regulation.
Cheng et al. [18]	2024	Taiwan	Article	PM2.5 exposure disrupts colon health by inducing inflammation, dysbiosis, epithelial hyperproliferation, autophagy dysregulation, and lysosomal damage, as evidenced by mouse models, human colon cell studies, and serum IL-8 correlations, underscoring its role in inflammatory bowel disease-like conditions.
Di Renzo et al. [131]	2024	Italy	Review	The exposome framework links chronic exposure to air pollution, socioeconomic disparities, lifestyle factors, gut microbiota alterations, and persistent toxicants to low-grade inflammation and non-communicable disease risk, highlighting the need for interdisciplinary research and precision medicine to improve prevention and intervention strategies.
Zhou et al. [20]	2024	UK	Article	Air pollution, including PM2.5, PM10, NOx, and PM2.5 absorbance, is linked to multiple sclerosis, AD, PTSD, and brain structural changes. Gut microbiota, such as *Lentisphaerae, Senegalimassilia*, and *C. inoculum*, mediate these effects. This highlights the gut-brain axis’s role in air pollution-induced neurotoxicity and the need for targeted interventions to mitigate environmental risks.
Bhardwaj et al. [132]	2024	India	Review	Environmental pollutants, including air pollution, chemicals, heavy metals, pesticides, and antibiotics, disrupt gut microbiota balance and impair metabolic, immune, and neurological functions. Further research on GM's role in pollutant-induced health effects is needed to develop effective mitigation strategies and safeguard public health.
Dosh et al. [133]	2024	Lebanon	Review	Gut microbiota plays a critical role in cardiovascular diseases, particularly atherosclerosis, by modulating immune responses and inflammation through metabolites, with dysbiosis exacerbating disease and probiotics offering therapeutic potential to restore balance and reduce inflammation, emphasizing the need for further research into gut-derived metabolic pathways for cardiovascular health.
Chang et al. [134]	2024	USA	Article	UFP inhalation for 10 weeks induces significant shifts in gut microbiome composition, increasing richness and evenness without immediate intestinal inflammation, with alterations preceding inflammatory markers, suggesting a delayed inflammatory response and highlighting the need for further research on the long-term health implications of environmental pollutants on gut health.
Liu et al. [16]	2024	China	Article	Oil mist particulate matter (OMPM) exposure disrupts immune homeostasis by increasing visceral fat, inflammatory cytokines, and gut microbiota imbalances, reducing SCFA production and downregulating the anti-inflammatory receptor GPR43, which activates the TLR4-NF-κB pathway in white adipose tissue, promoting inflammation and contributing to metabolic and inflammatory diseases, highlighting the need for regulatory actions to mitigate environmental exposure.
Zhang et al. [135]	2024	China	Review	Environmental and lifestyle factors, including pollution, stress, poor diet, sleep disturbances, inactivity, smoking, and caffeine, influence AD progression by affecting microglial function, with unhealthy habits increasing susceptibility and healthier choices potentially offering neuroprotection, emphasizing the need for further research on microglial mechanisms and therapeutic strategies.
Zhang et al. [136]	2024	USA	Article	Chronic OP exposure in humans, particularly among those with Parkinson’s disease, was linked to a sparser predicted metagenome and alterations in microbial abundance. These alterations influenced 22 genera and 34 metabolic pathways, including cellular respiration, bacterial wall biosynthesis, and vitamin B1 and B6 synthesis. This underscores the potential impact of long-term OP exposure on gut microbiome composition and metabolism.
Belloumi et al. [137]	2024	Spain	Article	The inclusion of 20% dried olive cake (OC) in pig diets led to significant changes in gut microbiota, with increases in health-promoting bacteria like *Planctomycota* and *Allisonella*, higher SCFA concentrations, and improved energy digestibility. This suggests that OC positively modulates gut microbiota and fermentation processes without affecting growth performance or gas emissions.
Wang et al. [12]	2024	China	Article	Gut microbiota species such as *Bifidobacteriales, Bifidobacteriaceae*, and *Ruminiclostridium9* were negatively associated with body fat percentage, while *Olsenella* and the metabolite valine were positively linked to it, suggesting that gut microbiota and metabolites play a role in obesity-related conditions and may be targets for therapeutic interventions.
Dehghani et al. [138]	2024	Netherlands	Article	Postnatal synbiotic supplementation reduced lung resistance, eosinophil counts, serum IgE/IgG1 levels, and promoted beneficial gut bacteria like *Bifidobacterium* and *Akkermansia*, suggesting its potential to mitigate allergic asthma symptoms in offspring exposed to environmental cigarette smoke and house-dust-mite challenges.
Dahiya et al. [139]	2024	India	Review	Exposure to heavy metals disrupts the gut microbiome, but probiotics, particularly with exopolysaccharides, help mitigate metal toxicity through biosorption, bioaccumulation, and biotransformation. Thus, probiotics offer a promising strategy for reducing heavy metal exposure and improving gut health.
Campolim et al. [140]	2024	Brazil	Short Survey	PM2.5 exposure promotes inflammation, disrupts gut microbiota, and exacerbates obesity, AD, and type 2 diabetes by triggering the TLR4 signaling pathway. This leads to leptin resistance, amyloid plaque deposition, and tau hyperphosphorylation, highlighting air pollution as a critical public health concern.
Utembe and Kamng’ona [141]	2024	South Africa	Review	Lung microbiota dysbiosis, influenced by inhaled pollutants and gut-lung interactions, is linked to respiratory diseases like asthma, COPD, lung cancer, and IPF. It has potential neurological impacts via the lung-brain axis, though causal mechanisms require further research.
Cruells et al. [142]	2024	Spain	Article	Prenatal and postnatal exposure to air pollution, especially NO2, reduces infant gut microbiota diversity and alters bacterial populations. Green spaces influence microbial composition and may offer protective effects, necessitating further research on long-term health impacts.
Mazumder and Hussain [143]	2024	USA	Review	Air pollution disrupts lung and gut microbiomes, causing dysbiosis, systemic inflammation, and epithelial barrier damage. These conditions exacerbate pulmonary and systemic diseases, while microbiome-based interventions may offer therapeutic potential.
Dai et al. [144]	2024	China	Article	Long-term PM2.5 exposure disrupts the lung-gut axis in male Balb/c mice, causing lung inflammation, emphysema, and altered metabolites, while shifting gut microbiota and metabolites, and affecting lung microbiota, all mediated by pathways like tryptophan metabolism and serotonergic signaling, underscoring its impact on respiratory and GI health.
Khandayataray and Murthy [145]	2024	India	Review	Environmental pollutants contribute to AD through neurotoxic mechanisms, and diets like the Mediterranean, DASH, and MIND diets offer promising preventive strategies by reducing oxidative stress, inflammation, and improving gut microbiota, emphasizing the need for research on diet-environment interactions to mitigate AD risk.
Wong et al. [146]	2024	China	Review	Char, a carbon-rich material produced from biomass pyrolysis, enhances water quality, reduces pollutants, improves fish health, and supports growth and intestinal health when used as a feed supplement. However, further research is required to understand its mechanisms for pollutant removal, transport within aquatic organisms, and effects on gut microbiota.
Liu et al. [147]	2024	China	Article	Air pollution, particularly PM2.5, PM10, PM2.5 absorbance, and NOx, is linked to cardiovascular and metabolic diseases. Gut microbiota, especially Ruminococcaceae-UCG003, mediates 7.8% of PM2.5's effect on type 2 diabetes, suggesting that air quality improvement and microbiome modulation could help mitigate pollution-induced health risks.
Hameed et al. [148]	2024	USA	Review	Emerging stroke risk factors, including air pollution, gut microbiota imbalances, high altitude, and systemic infections, contribute to the incidence of strokes. Modifiable interventions, such as environmental policies, microbiome modulation, and infection prevention, offer potential strategies to reduce global stroke rates and enhance public health.
Darma et al. [10]	2024	Indonesia	Review	Air pollution alters gut microbiota composition, potentially linking dysbiosis to asthma in children. Probiotics, prebiotics, and synbiotics show promise in improving gut health, enhancing immune responses, and preventing infections, though further research is needed to fully understand their effectiveness.
Keerthy et al. [149]	2024	USA	Article	In utero exposure to PAHs was associated with changes in the meconium microbiome, with high PAH exposure linked to decreased alpha diversity and low/medium exposure to increased diversity. This suggests potential long-term impacts on gut colonization and child health, though further research is needed.
Qiu et al. [150]	2024	China	Article	Maternal PM2.5 exposure was linked to higher depression scores and gut microbiota alterations, with reduced *Enterococcus* and *Enterobacter* levels, and *Enterococcus* was found to mediate a significant portion of the relationship between PM2.5 exposure and antenatal depression, suggesting its potential protective role.
Du et al. [151]	2024	China	Article	Gut microbiota, blood metabolites, and host genes influence lung cancer development, with *Bacteroides clarus* increasing risk, *Eubacteriaceae* providing protection, key metabolites like cystine and propionylcarnitine acting as risk modifiers, and metabolic pathways such as glutathione and glutamate metabolism playing crucial roles.
Li et al. [152]	2024	China	Article	PM2.5 exposure was linked to an increased risk of ASD, ADHD, schizophrenia, and AD, with gut microbiota, particularly *Lachnospiraceae* and *Barnesiella*, partially mediating the effects on ADHD and schizophrenia, highlighting air pollution's role in neuropsychiatric disorders and the need for stricter air quality regulations.
Zha et al. [153]	2024	China	Article	Foodborne PES MNP disrupted the gut-liver axis, causing gut microbiota dysbiosis, liver injury, and altered liver transcriptomics, while airborne PES MNP impaired the lung microbiota-lung axis, leading to lung damage and metabolic disturbances, with both exposures reducing antioxidant activity in serum, underscoring the need for stricter PES MNP management to mitigate health risks.
Chen et al. [154]	2024	Taiwan	Article	Maternal diesel exhaust particle exposure led to lung injury, increased macrophage activity, elevated cytokine levels in offspring, and gut microbiota dysbiosis by postnatal day 7, while maternal gut microbiota remained unchanged, highlighting the broader impact of air pollution on fetal health.
Padhi et al. [155]	2024	United States	Article	Environmental chemical pollutants, including heavy metals, pesticides, air pollution, and industrial chemicals, disrupt the gut microbiome, potentially contributing to neurotoxicity by altering microbial balance and influencing neurological health, emphasizing the need for further research and intervention strategies.
Guilloteau et al. [156]	2024	France	Article	Prenatal air pollution exposure causes sex-specific intestinal alterations in mice, with males experiencing structural damage and immune imbalance while females exhibit gut microbiota dysbiosis and gene expression changes, potentially leading to long-term health consequences.
Santos-Silva et al. [157]	2024	Brazil	Article	Cadmium and mercury contamination in the Amazon rainforest altered the bacterial microbiota of *Brachyurodesmus albus*, disrupting microbial diversity, metabolic pathways, and antimicrobial resistance genes, while Cd- and Hg-resistant bacteria demonstrated potential for bioremediation by enhancing plant tolerance.
Gouider et al. [158]	2024	Tunisia	Article	Environmental and lifestyle factors such as low sun exposure, vitamin D deficiency, January births, Epstein-Barr virus infection, smoking, obesity, air pollution, and gut microbiota alterations contribute to multiple sclerosis progression and severity, with comorbidities like cardiovascular disease, epilepsy, and depression accelerating disability.
Pan et al. [159]	2024	China	Article	Indoor PM2.5 exposure accelerates biological aging in schizophrenia patients through components like thallium, chromium, and manganese, with serum glycerophospholipid metabolites and gut microbiota, particularly *Dorea* and *Blautia*, mediating these effects, highlighting the need to address indoor pollution in vulnerable populations.
Shao et al. [160]	2024	China	Article	PM2.5 exposure disrupts gut microbiota, leading to glucose metabolic abnormalities, but modifying the microbiota or supplementing with acetate can alleviate these effects and improve glucose metabolism.
Sugden and Merlo [161]	2024	USA	Review	The review emphasizes the interconnected impact of climate change, air pollution, and rising depression rates, suggesting that plant-based diets can improve mental health, enhance gut microbiota, and address environmental challenges, offering a sustainable solution for both personal and global well-being.
Zaltman et al. [162]	2024	Brazil	Review	The review highlights a potential link between air pollution and IBD, suggesting it may contribute to inflammation, microbiota imbalances, and impaired colonic function. However, it stresses the need for further research to clarify this complex relationship and its impact on disease prevention and management.
Wang et al. [163]	2025	China	Article	Subchronic exposure to chloroform in female Kunming mice significantly disrupted hematological parameters, caused histopathological changes in cecal tissues, and altered the gut microbiome composition and metabolic balance in the cecum, with a strong correlation between microbiome changes and differentially expressed metabolites.

Discussion

The findings of this scoping review underscore the profound and complex impact of air pollution on gut microbiota and its far-reaching consequences on human health. The gut microbiota, often referred to as the "second genome," plays a crucial role in maintaining homeostasis through its involvement in nutrient metabolism, immune modulation, and protection against pathogens. The disruption of this delicate microbial balance by environmental pollutants, particularly PM, VOCs, and heavy metals, represents a growing public health concern. The reviewed studies demonstrate that air pollution-induced gut dysbiosis primarily affects gastrointestinal health. It extends its impact to respiratory, neurological, metabolic, and cardiovascular health through interconnected biological pathways, including the gut-lung and the gut-brain axes.

Mechanisms of Air Pollution-Induced Gut Dysbiosis

A primary way that air pollution affects gut health is through the direct contact between inhaled pollutants and the gut lining. The mucociliary route allows PM and toxins inhaled into the lungs to be transported to the digestive tract, where they come into contact with the intestinal lining. This direct exposure can lead to increased intestinal permeability, often referred to as "leaky gut," which allows harmful substances and microbial endotoxins to enter the bloodstream and trigger systemic inflammation. Ammonia exposure induces gut dysbiosis through TLR4-mediated inflammation, highlighting how immune signaling pathways are activated in response to air pollutants [13]. Similarly, PM2.5 exposure leads to epithelial hyperproliferation, dysregulation of autophagy, and lysosomal damage in colonic cells, further exacerbating inflammation and microbial imbalance [18].

Beyond direct interactions, pollutants such as heavy metals, polycyclic aromatic hydrocarbons (PAHs), and VOCs have been shown to alter the gut microbiota's composition and function. Vehicle exhaust exposure shortened lifespan and impaired locomotion in *Drosophila* by disrupting gut microbiota and increasing intestinal permeability [14]. Liu et al. [16] reported that OMPM exposure reduces the production of SCFAs, critical for maintaining gut barrier integrity and immune function. Reduced SCFA production subsequently activates the TLR4-NF-κB inflammatory pathway, leading to increased visceral fat accumulation and contributing to metabolic dysfunction.

The Gut-Lung Axis and Respiratory Health

The gut-lung axis is increasingly recognized as a critical pathway through which air pollution affects respiratory health. Lung immune responses are influenced by the composition of the gut microbiome, which produces microbial metabolites such as SCFAs. Disruption of gut microbiota balance has been linked to increased susceptibility to respiratory infections, asthma, COPD, and lung cancer. Gupta et al. [17] reported that *L. plantarum *mitigates pulmonary inflammation caused by PM exposure through its antioxidant activity and capacity to regulate gut-lung axis communication. Similarly, Cheng et al. [18] demonstrated that PM2.5 exposure induces colonic inflammation and dysbiosis, subsequently increasing pro-inflammatory cytokine production and exacerbating pulmonary damage.

Furthermore, specific microbial strains have been identified as playing a protective role in maintaining lung health. Simon et al. [9] highlighted that *B. megaterium* (Renuspore®) exhibits detoxifying effects against heavy metals and pollutants, producing beneficial metabolites such as SCFAs that counteract inflammation and restore gut-lung balance. This suggests that probiotic interventions targeting gut health could play a protective role in mitigating air pollution-related respiratory diseases.

The Gut-Brain Axis and Neurological Implications

The gut-brain axis also emerges as a key mediator of air pollution's impact on neurological health. The central nervous system communicates with the gut via the vagus nerve, allowing microbial metabolites to influence brain function and behavior. Disruption of this communication by air pollution-induced gut dysbiosis has been linked to cognitive decline, neuroinflammation, and psychiatric disorders. Yuan et al. [19] found that indoor air pollution in rural China was associated with cognitive impairment in 67.5% of elderly residents, particularly among women. The altered gut microbiota profile, characterized by a reduction in *Faecalibacterium* and an increase in *Escherichia*-*Shigella* levels, was accompanied by increased systemic inflammation and oxidative stress.

Zhou et al. [20] further demonstrated that air pollution exposure is linked to brain structural changes and the development of neurological diseases, such as Alzheimer's disease and multiple sclerosis. Specific gut microbiota shifts, such as an increase in *Lentisphaerae* and *Clostridium innocuum*, were found to mediate these effects, underscoring the critical role of gut microbiota in neurodegenerative processes. Moreover, Sun et al. [21] demonstrated that the OXTR gene polymorphism affects the relationship between air pollution and gut microbiota, underscoring the need for personalized approaches to mitigate cognitive and emotional disturbances associated with environmental pollution.

Metabolic and Cardiovascular Implications

Air pollution's impact on gut microbiota extends to metabolic and cardiovascular health. The gut microbiota regulates lipid metabolism, systemic inflammation, and glucose homeostasis, processes that are directly affected by exposure to air pollutants. PM2.5 exposure increases insulin resistance through gut microbiota-driven changes in sphingolipid metabolism [15]. PM exposure increases visceral fat accumulation and pro-inflammatory cytokine production, thereby promoting the development of metabolic syndrome and cardiovascular diseases [16].

Specific microbial taxa have been linked to metabolic health outcomes. Wang et al. [12] reported that PM exposure reduces the abundance of *Bifidobacterium* and increases pro-inflammatory bacteria such as *Proteobacteria* and *Enterobacteriaceae*, exacerbating obesity and metabolic syndrome. Similarly, Wang et al. [12] found that gut microbiota composition, including an increase in *Bifidobacteriales* and a decrease in *Olsenella*, was closely linked to body fat percentage and metabolic dysfunction.

Potential Interventions and Protective Strategies

Several protective strategies emerged from the reviewed studies. Probiotics, prebiotics, and synbiotics have shown promise in restoring gut microbiota balance and mitigating dysbiosis induced by air pollution. *L. plantarum* improved gut and pulmonary health in PM-exposed models [17]. Liu et al. [9] highlighted the detoxifying effects of *B. megaterium* on heavy metals and pollutants, improving gut microbiota resilience. Dietary interventions, such as those containing fish-derived proteins and fruit-derived biomolecules, also show potential in reducing oxidative stress and restoring gut homeostasis [8].

Despite the consistent evidence linking air pollution to gut microbiota dysbiosis, this review has several limitations: (1) The included studies used varying methodologies, including observational, experimental, and review-based approaches. This heterogeneity makes it difficult to compare results and draw unified conclusions. While some studies used animal models, others relied on human data, which may limit the applicability of findings across species and populations. (2) Most studies were cross-sectional or short-term, which limits the ability to establish causality between air pollution exposure and gut dysbiosis. Long-term studies are necessary to evaluate the cumulative effects of chronic pollution exposure on gut health. (3) Most studies were conducted in East Asia and North America, which limits the generalizability of the findings to other regions, particularly LMICs, where air pollution levels are often higher. (4) Environmental, dietary, and genetic factors may have contributed to the observed changes in gut microbiota. However, many studies did not control for these variables, introducing the possibility of residual confounding. (5) While PM2.5 and other common pollutants were extensively studied, fewer studies examined the effects of specific pollutants, such as heavy metals, VOCs, and microplastics, on gut microbiota composition. More targeted research is needed on the differential effects of individual pollutants. (6) Our search was comprehensive; however, we didn't have the chance to check all the databases.

## Conclusions

This scoping review highlights the significant impact of air pollution on gut microbiota and its broader health consequences. Exposure to PM, heavy metals, and VOCs has been frequently associated with disruption in gut microbial balance, leading to increased intestinal permeability, inflammation, and immune dysregulation. These changes contribute to respiratory diseases (e.g., asthma, COPD), metabolic disorders (e.g., obesity, insulin resistance), and neurological conditions (e.g., Alzheimer's disease, cognitive decline) through the gut-lung and gut-brain axes. Promising interventions include prebiotics, probiotics, and synbiotics, which have shown potential in restoring gut balance and reducing inflammation. Dietary interventions such as fish-derived proteins and fruit-derived biomolecules also demonstrate protective effects. However, research limitations, including study heterogeneity, lack of longitudinal data, and geographical bias, restrict the generalizability of findings.

Future research should focus on longitudinal studies, include diverse populations, and utilize integrative multi-omics techniques to understand causality and mechanisms. These insights are essential for developing personalized treatments and informing public health policies that aim to lower the health impacts of environmental pollution through microbiome modulation.

## References

[1] Vilcins D, Christofferson RC, Yoon JH, Nazli SN, Sly PD, Cormier SA, Shen G (2024). Updates in air pollution: current research and future challenges. Ann Glob Health.

[2] Berman JD (2024). Air pollution and health - new advances for an old public health problem. JAMA Netw Open.

[3] Salim SY, Kaplan GG, Madsen KL (2014). Air pollution effects on the gut microbiota: a link between exposure and inflammatory disease. Gut Microbes.

[4] GBD 2021 HAP Collaborators (2025). Global, regional, and national burden of household air pollution, 1990-2021: a systematic analysis for the Global Burden of Disease Study 2021. Lancet.

[5] Gupta N, Yadav VK, Gacem A (2022). Deleterious effect of air pollution on human microbial community and bacterial flora: a short review. Int J Environ Res Public Health.

[6] Rio P, Gasbarrini A, Gambassi G, Cianci R (2024). Pollutants, microbiota and immune system: frenemies within the gut. Front Public Health.

[7] Habotta OA, Dawood MA, Kari ZA, Tapingkae W, Van Doan H (2022). Antioxidative and immunostimulant potential of fruit derived biomolecules in aquaculture. Fish Shellfish Immunol.

[8] Liu D, Ren Y, Zhong S, Xu B (2024). New insight into utilization of fish by-product proteins and their skin health promoting effects. Mar Drugs.

[9] Simon A, Colom J, Mazhar S, Khokhlova E, Deaton J, Rea K (2023). Bacillus megaterium Renuspore(®) as a potential probiotic for gut health and detoxification of unwanted dietary contaminants. Front Microbiol.

[10] Darma A, Dewi DK, Chandra DN, Basrowi RW, Khoe LC, Pratiwi D (2024). The role of prebiotic, probiotic, and synbiotic in gut microbiota and gut permeability in children affected by air pollution. Curr Nutr Food Sci.

[11] Tricco AC, Lillie E, Zarin W (2018). PRISMA extension for scoping reviews (PRISMA-ScR): checklist and explanation. Ann Intern Med.

[12] Wang X, Lu C, Li X, Ye P, Ma J, Chen X (2024). Exploring causal effects of gut microbiota and metabolites on body fat percentage using two-sample Mendelian randomization. Diabetes Obes Metab.

[13] Zhou Y, Zhao X, Zhang M, Feng J (2022). Gut microbiota dysbiosis exaggerates ammonia-induced tracheal injury Via TLR4 signaling pathway. Ecotoxicol Environ Saf.

[14] Li Y, Pan L, Li P (2022). Microbiota aggravates the pathogenesis of Drosophila acutely exposed to vehicle exhaust. Heliyon.

[15] Zhao L, Fang J, Tang S (2022). PM2.5 and serum metabolome and insulin resistance, potential mediation by the gut microbiome: a population-based panel study of older adults in China. Environ Health Perspect.

[16] Liu H, Nie H, Shi Y (2024). Oil mistparticulate matter exposure induces hyperlipidemia-related inflammation via microbiota/ SCFAs/GPR43 axis inhibition and TLR4/NF-κB activation. Environ Pollut.

[17] Gupta N, Abd El-Gawaad NS, Osman Abdallah SA, Al-Dossari M (2023). Possible modulating functions of probiotic Lactiplantibacillus plantarum in particulate matter-associated pulmonary inflammation. Front Cell Infect Microbiol.

[18] Cheng HJ, Hsu WL, Lin P (2024). Involvement of autophagy and gut dysbiosis in ambient particulate matter-induced colonic inflammation. Ecotoxicol Environ Saf.

[19] Yuan J, Tan H, Cheng Y (2024). Air particulate pollution exposure associated with impaired cognition via microbiota gut-brain axis: an evidence from rural elderly female in northwest China. Environ Sci Pollut Res Int.

[20] Zhou X, Guo Z, Ling Y (2024). Causal effect of air pollution on the risk of brain health and potential mediation by gut microbiota. Ecotoxicol Environ Saf.

[21] Sun Y, Deng G, Fan J, Feng F, Ge Q, Song Y, Kang X (2022). Associations of air PM(2.5) level with gut microbiota in Chinese Han preschoolers and effect modification by oxytocin receptor gene polymorphism. Environ Res.

[22] Yaohan W, Nannan H, Bin L, Hanqing C, Xiaobo L, Rui C (2024). Research progress on regulation of gut microbiota abundance induced by ambient particulate matter exposure. J Environ Occup Med.

[23] Bailey MJ, Naik NN, Wild LE, Patterson WB, Alderete TL (2020). Exposure to air pollutants and the gut microbiota: a potential link between exposure, obesity, and type 2 diabetes. Gut Microbes.

[24] Lin LZ, Chen JH, Yu YJ, Dong GH (2023). Ambient air pollution and infant health: a narrative review. EBioMedicine.

[25] Kozyrskyj AL, Bahreinian S, Azad MB (2011). Early life exposures: impact on asthma and allergic disease. Curr Opin Allergy Clin Immunol.

[26] Haahtela T, Holgate S, Pawankar R (2013). The biodiversity hypothesis and allergic disease: World Allergy Organization position statement. World Allergy Organ J.

[27] Wiernsperger N (2014). Treatment strategies for fatty liver diseases. Rev Recent Clin Trials.

[28] Martin TD, Chan SS, Hart AR (2015). Environmental factors in the relapse and recurrence of inflammatory bowel disease: a review of the literature. Dig Dis Sci.

[29] Abegunde AT, Muhammad BH, Bhatti O, Ali T (2016). Environmental risk factors for inflammatory bowel diseases: evidence based literature review. World J Gastroenterol.

[30] Adar SD, Huffnagle GB, Curtis JL (2016). The respiratory microbiome: an underappreciated player in the human response to inhaled pollutants?. Ann Epidemiol.

[31] Rosenfeld CS (2017). Gut dysbiosis in animals due to environmental chemical exposures. Front Cell Infect Microbiol.

[32] Chotirmall SH, Gellatly SL, Budden KF (2017). Microbiomes in respiratory health and disease: an Asia-Pacific perspective. Respirology.

[33] Alderete TL, Jones RB, Chen Z (2018). Exposure to traffic-related air pollution and the composition of the gut microbiota in overweight and obese adolescents. Environ Res.

[34] Mutlu EA, Comba IY, Cho T (2018). Inhalational exposure to particulate matter air pollution alters the composition of the gut microbiome. Environ Pollut.

[35] Vallès Y, Francino MP (2018). Air pollution, early life microbiome, and development. Curr Environ Health Rep.

[36] Ananthakrishnan AN, Bernstein CN, Iliopoulos D (2018). Environmental triggers in IBD: a review of progress and evidence. Nat Rev Gastroenterol Hepatol.

[37] Saeed M, Arain MA, Naveed M (2018). Yucca schidigera can mitigate ammonia emissions from manure and promote poultry health and production. Environ Sci Pollut Res Int.

[38] Kim JE, Kim HS (2019). Microbiome of the skin and gut in atopic dermatitis (AD): understanding the pathophysiology and finding novel management strategies. J Clin Med.

[39] Sbihi H, Boutin RC, Cutler C, Suen M, Finlay BB, Turvey SE (2019). Thinking bigger: how early-life environmental exposures shape the gut microbiome and influence the development of asthma and allergic disease. Allergy.

[40] Liu T, Chen X, Xu Y (2019). Gut microbiota partially mediates the effects of fine particulate matter on type 2 diabetes: evidence from a population-based epidemiological study. Environ Int.

[41] Curciarello R, Canziani KE, Docena GH, Muglia CI (2019). Contribution of non-immune cells to activation and modulation of the intestinal inflammation. Front Immunol.

[42] Ristori MV, Quagliariello A, Reddel S, Ianiro G, Vicari S, Gasbarrini A, Putignani L (2019). Autism, gastrointestinal symptoms and modulation of gut microbiota by nutritional interventions. Nutrients.

[43] Dempsey JL, Little M, Cui JY (2019). Gut microbiome: an intermediary to neurotoxicity. Neurotoxicology.

[44] Li X, Sun H, Li B (2019). Probiotics ameliorate colon epithelial injury induced by ambient ultrafine particles exposure. Adv Sci (Weinh).

[45] Roslund MI, Rantala S, Oikarinen S (2019). Endocrine disruption and commensal bacteria alteration associated with gaseous and soil PAH contamination among daycare children. Environ Int.

[46] Fox M, Knorr DA, Haptonstall KM (2019). Alzheimer's disease and symbiotic microbiota: an evolutionary medicine perspective. Ann N Y Acad Sci.

[47] Turkalj M (2019). Respiratory and gut microbiota in allergy and asthma. Cent Eur J Paediatr.

[48] Frati F, Salvatori C, Incorvaia C, Bellucci A, Di Cara G, Marcucci F, Esposito S (2018). The role of the microbiome in asthma: the gut-lung axis. Int J Mol Sci.

[49] Liu W, Zhou Y, Yong Li (2020). Effects of PM(2.5) exposure during gestation on maternal gut microbiota and pregnancy outcomes. Chemosphere.

[50] Fitch MN, Phillippi D, Zhang Y (2020). Effects of inhaled air pollution on markers of integrity, inflammation, and microbiota profiles of the intestines in Apolipoprotein E knockout mice. Environ Res.

[51] Wan-jun W, Zhou-Zhou L, Yan-yi X (2020). Advances on mechanisms of abnormal glucose metabolism induced by ambient fine particulate matters. J Environ Occup Med.

[52] Feng J, Cavallero S, Hsiai T, Li R (2020). Impact of air pollution on intestinal redox lipidome and microbiome. Free Radic Biol Med.

[53] Liu W, Zhou Y, Qin Y, Yu L, Li R, Chen Y, Xu Y (2020). Quercetin intervention alleviates offspring’s oxidative stress, inflammation, and tight junction damage in the colon induced by maternal fine particulate matter (PM2.5) exposure through the reduction of Bacteroides. Nutrients.

[54] Huang R, Ju Z, Zhou PK (2020). A gut dysbiotic microbiota-based hypothesis of human-to-human transmission of non-communicable diseases. Sci Total Environ.

[55] Wang H, Adamcakova-Dodd A, Flor S (2020). Comprehensive subchronic inhalation toxicity assessment of an indoor school air mixture of PCBs. Environ Sci Technol.

[56] Donovan C, Liu G, Shen S (2020). The role of the microbiome and the NLRP3 inflammasome in the gut and lung. J Leukoc Biol.

[57] Liberti A, Bertocci I, Pollet A (2020). An indoor study of the combined effect of industrial pollution and turbulence events on the gut environment in a marine invertebrate. Mar Environ Res.

[58] Leyva-López N, Lizárraga-Velázquez CE, Hernández C, Sánchez-Gutiérrez EY (2020). Exploitation of agro-industrial waste as potential source of bioactive compounds for aquaculture. Foods.

[59] Dujardin CE, Mars RA, Manemann SM, Kashyap PC, Clements NS, Hassett LC, Roger VL (2020). Impact of air quality on the gastrointestinal microbiome: a review. Environ Res.

[60] Narla S, Silverberg JI (2020). The role of environmental exposures in atopic dermatitis. Curr Allergy Asthma Rep.

[61] DeKruyff RH, Zhang W, Nadeau KC, Leung DY, Wills-Karp M (2020). Summary of the Keystone Symposium "Origins of allergic disease: microbial, epithelial and immune interactions," March 24-27, Tahoe City, California. J Allergy Clin Immunol.

[62] Fouladi F, Bailey MJ, Patterson WB (2020). Air pollution exposure is associated with the gut microbiome as revealed by shotgun metagenomic sequencing. Environ Int.

[63] Zheng P, Zhang B, Zhang K, Lv X, Wang Q, Bai X (2020). The impact of air pollution on intestinal microbiome of asthmatic children: a panel study. Biomed Res Int.

[64] Di Tommaso N, Gasbarrini A, Ponziani FR (2021). Intestinal barrier in human health and disease. Int J Environ Res Public Health.

[65] van den Brule S, Rappe M, Ambroise J (2021). Diesel exhaust particles alter the profile and function of the gut microbiota upon subchronic oral administration in mice. Part Fibre Toxicol.

[66] Cabrera C, Vicens P, Torrente M (2021). Modifiable risk factors for dementia: the role of gut microbiota. Curr Alzheimer Res.

[67] Vignal C, Guilloteau E, Gower-Rousseau C, Body-Malapel M (2021). Review article: epidemiological and animal evidence for the role of air pollution in intestinal diseases. Sci Total Environ.

[68] Yang Y, Yang C, Lei Z (2021). Cigarette smoking exposure breaks the homeostasis of cholesterol and bile acid metabolism and induces gut microbiota dysbiosis in mice with different diets. Toxicology.

[69] Anwar H, Iftikhar A, Muzaffar H (2021). Biodiversity of gut microbiota: impact of various host and environmental factors. Biomed Res Int.

[70] Wang G, McDonald VM (2021). Contemporary concise review 2020: asthma. Respirology.

[71] Nowak A, Szczuka D, Górczyńska A, Motyl I, Kręgiel D (2021). Characterization of Apis mellifera gastrointestinal microbiota and lactic acid bacteria for honeybee protection - a review. Cells.

[72] Cui G, Liu H, Xu G, Laugsand JB, Pang Z (2021). Exploring links between industrialization, urbanization, and Chinese inflammatory bowel disease. Front Med (Lausanne).

[73] Fournier E, Etienne-Mesmin L, Grootaert C, Jelsbak L, Syberg K, Blanquet-Diot S, Mercier-Bonin M (2021). Microplastics in the human digestive environment: a focus on the potential and challenges facing in vitro gut model development. J Hazard Mater.

[74] Lu D, Hai W, Yixiao P, Xiaokui G, Chang L (2021). Correlation between particulate matter and intestinal microflora and intestinal inflammation: research progress. Chin J Microecol.

[75] Earp E, Tsianou Z, Grindlay DJ, Rogers NK, Olabi B (2021). What's new in atopic eczema? An analysis of systematic reviews published in 2019. Part 1: risk factors and prevention. Clin Exp Dermatol.

[76] Paul AK, Paul A, Jahan R (2021). Probiotics and amelioration of rheumatoid arthritis: Significant roles of Lactobacillus casei and Lactobacillus acidophilus. Microorganisms.

[77] Sheina NI, Budanova EV, Kolesnikova VV, Mjalina LI, Sazonova LI (2021). The intestinal microbiota of rats under the influence of biotechnological strains of microorganisms from various taxonomic groups. Hyg Sanit.

[78] Stefanovic N, Irvine AD, Flohr C (2021). The role of the environment and exposome in atopic dermatitis. Curr Treat Options Allergy.

[79] Wang Y, Dykes G (2021). Gut microbiota as a link between modern lifestyle and Alzheimer’s disease. Curr Aging Sci.

[80] Juanola O, Martínez-López S, Francés R, Gómez-Hurtado I (2021). Non-alcoholic fatty liver disease: metabolic, genetic, epigenetic and environmental risk factors. Int J Environ Res Public Health.

[81] Alenius H, Sinkko H, Moitinho-Silva L (2021). The power and potential of BIOMAP to elucidate host-microbiome interplay in skin inflammatory diseases. Exp Dermatol.

[82] Li A, Wang Y, He Y (2021). Environmental fluoride exposure disrupts the intestinal structure and gut microbial composition in ducks. Chemosphere.

[83] Vari HK, Roslund MI, Oikarinen S (2021). Associations between land cover categories, gaseous PAH levels in ambient air and endocrine signaling predicted from gut bacterial metagenome of the elderly. Chemosphere.

[84] Liyi Z, Yuhan Z, Yunhui Z (2021). Association between PM2.5 exposure during pregnancy and meconium microbiome of newborns. J Environ Occup Med.

[85] Pulliero A, Traversi D, Franchitti E, Barchitta M, Izzotti A, Agodi A (2021). The interaction among microbiota, epigenetic regulation, and air pollutants in disease prevention. J Pers Med.

[86] Santa K (2022). Grape phytochemicals and vitamin D in the alleviation of lung disorders. Endocr Metab Immune Disord Drug Targets.

[87] Basarkar V, Govardhane S, Shende P (2022). Multifaceted applications of genetically modified micro-organisms: a biotechnological revolution. Curr Pharm Des.

[88] Sommer AJ, Peters A, Rommel M (2022). A randomization-based causal inference framework for uncovering environmental exposure effects on human gut microbiota. PLoS Comput Biol.

[89] Li A, Wang Y, Hao J (2022). Long-term hexavalent chromium exposure disturbs the gut microbial homeostasis of chickens. Ecotoxicol Environ Saf.

[90] Bailey MJ, Holzhausen EA, Morgan ZE (2022). Postnatal exposure to ambient air pollutants is associated with the composition of the infant gut microbiota at 6-months of age. Gut Microbes.

[91] Dutta M, Weigel KM, Patten KT (2022). Chronic exposure to ambient traffic-related air pollution (TRAP) alters gut microbial abundance and bile acid metabolism in a transgenic rat model of Alzheimer's disease. Toxicol Rep.

[92] Mbareche H, Veillette M, Dion-Dupont V (2022). Microbial composition of bioaerosols in indoor wastewater treatment plants. Aerobiologia.

[93] Mundula T, Russo E, Curini L, Giudici F, Piccioni A, Franceschi F, Amedei A (2022). Chronic systemic low-grade inflammation and modern lifestyle: the dark role of gut microbiota on related diseases with a focus on COVID-19 pandemic. Curr Med Chem.

[94] Phillippi DT, Daniel S, Pusadkar V (2022). Inhaled diesel exhaust particles result in microbiome-related systemic inflammation and altered cardiovascular disease biomarkers in C57Bl/6 male mice. Part Fibre Toxicol.

[95] Keulers L, Dehghani A, Knippels L (2022). Probiotics, prebiotics, and synbiotics to prevent or combat air pollution consequences: the gut-lung axis. Environ Pollut.

[96] Smith MM, Melrose J (2022). Xylan prebiotics and the gut microbiome promote health and wellbeing: potential novel roles for pentosan polysulfate. Pharmaceuticals (Basel).

[97] Malecki KMC, Nikodemova M, Schultz AA (2022). The Survey of the Health of Wisconsin (SHOW) Program: an infrastructure for advancing population health. Front Public Health.

[98] Pambianchi E, Pecorelli A, Valacchi G (2022). Gastrointestinal tissue as a "new" target of pollution exposure. IUBMB Life.

[99] Filardo S, Di Pietro M, Protano C, Antonucci A, Vitali M, Sessa R (2022). Impact of air pollution on the composition and diversity of human gut microbiota in general and vulnerable populations: a systematic review. Toxics.

[100] Gan Q, Ye W, Zhao X (2022). Mediating effects of gut microbiota in the associations of air pollutants exposure with adverse pregnancy outcomes. Ecotoxicol Environ Saf.

[101] Wang B, Zhang SQ, Dong JL (2022). Ambient temperature structures the gut microbiota of zebrafish to impact the response to radioactive pollution. Environ Pollut.

[102] Mousavi SE, Delgado-Saborit JM, Adivi A, Pauwels S, Godderis L (2022). Air pollution and endocrine disruptors induce human microbiome imbalances: a systematic review of recent evidence and possible biological mechanisms. Sci Total Environ.

[103] Alharbi KS, Alenezi SK, Nasser S (2022). Microbiome in asthma. Microbiome in Inflammatory Lung Diseases.

[104] Dai S, Wang Z, Yang Y, Du P, Li X (2022). PM(2.5) induced weight loss of mice through altering the intestinal microenvironment: mucus barrier, gut microbiota, and metabolic profiling. J Hazard Mater.

[105] Fadlyana E, Soemarko DS, Endaryanto A (2022). The impact of air pollution on gut microbiota and children’s health: an expert consensus. Children (Basel).

[106] Boroujeni MH, Dehdashti B, Amin MH, Adibi P (2023). The possible effects of common air pollutants on the pathophysiology of functional gastrointestinal disorders: a narrative review. Int J Environ Health Eng.

[107] Wang J, Yan Y, Si H (2023). The effect of real-ambient PM2.5 exposure on the lung and gut microbiomes and the regulation of Nrf2. Ecotoxicol Environ Saf.

[108] Zhao L, Li B, Zhou L (2023). PM(2.5) exposure promotes asthma in aged Brown-Norway rats: implication of multiomics analysis. Ecotoxicol Environ Saf.

[109] Liu X, Wang X, Zhang P, Fang Y, Liu Y, Ding Y, Zhang W (2023). Intestinal homeostasis in the gut-lung-kidney axis: a prospective therapeutic target in immune-related chronic kidney diseases. Front Immunol.

[110] Chelu S, Kundnani NR, Chiriac V (2023). Prevention is better than cure: identifying and dealing with the key features involved in the prevention of gestational diabetes - a bird's eye view. Eur Rev Med Pharmacol Sci.

[111] Mazumder MH, Gandhi J, Majumder N (2023). Lung-gut axis of microbiome alterations following co-exposure to ultrafine carbon black and ozone. Part Fibre Toxicol.

[112] Goveas LC, Nayak S, Kumar PS (2023). Microplastics occurrence, detection and removal with emphasis on insect larvae gut microbiota. Mar Pollut Bull.

[113] Qiu T, Zang T, Fang Q (2023). Cumulative and lagged effects of varying-sized particulate matter exposure associates with toddlers' gut microbiota. Environ Pollut.

[114] Li H, Yang Y, Yang Y (2024). Multiomics was used to clarify the mechanism by which air pollutants affect chronic obstructive pulmonary disease: a human cohort study. Toxicology.

[115] Sadeghi A, Leddin D, Malekzadeh R (2023). Mini review: the impact of climate change on gastrointestinal health. Middle East J Dig Dis.

[116] Van Pee T, Nawrot TS, van Leeuwen R, Hogervorst J (2023). Ambient particulate air pollution and the intestinal microbiome; a systematic review of epidemiological, in vivo and, in vitro studies. Sci Total Environ.

[117] Barouki R, Samson M, Blanc EB (2023). The exposome and liver disease - how environmental factors affect liver health. J Hepatol.

[118] Aribi M, Mennechet FJ, Touil-Boukoffa C (2023). Editorial: the role of vitamin D as an immunomodulator. Front Immunol.

[119] Shen J, Liang B, Jin H (2023). The impact of microplastics on insect physiology and the indication of hormesis. TrAC Trends Anal Chem.

[120] Korbush MY, Serhiichuk TM, Yumyna YM, Borisova TO, Tolstanova GM (2023). The effect of particulate matter of natural and anthropogenic origin on growth indicators. Mikrobiol Zh.

[121] Dorofeyev A, Dorofeyeva A, Borysov A, Tolstanova G, Borisova T (2023). Gastrointestinal health: changes of intestinal mucosa and microbiota in patients with ulcerative colitis and irritable bowel syndrome from PM(2.5)-polluted regions of Ukraine. Environ Sci Pollut Res Int.

[122] Abdelkawi A, Sabbagh D, Hobi M, Pathak YV (2023). Effects of psychological, environmental, and physical stressors on the gut microbiota. Anxiety, Gut Microbiome, and Nutraceuticals.

[123] Singh SA, Suresh S, Vellapandian C (2023). Ozone-induced neurotoxicity: in vitro and in vivo evidence. Ageing Res Rev.

[124] Cheng TY, Chang CC, Luo CS, Chen KY, Yeh YK, Zheng JQ, Wu SM (2023). Targeting lung-gut axis for regulating pollution particle-mediated inflammation and metabolic disorders. Cells.

[125] Kim A, Zisman CR, Holingue C (2023). Influences of the immune system and microbiome on the etiology of ASD and GI symptomology of autistic individuals. Curr Top Behav Neurosci.

[126] Yao M, Guo X, Shao X, Wei Y, Zhang X, Wang H, Xu F (2023). Soluble dietary fiber from Prunus persica dregs alleviates gut microbiota dysfunction through lead excretion. Food Chem Toxicol.

[127] Ben-Azu B, Del Re EC, VanderZwaag J, Carrier M, Keshavan M, Khakpour M, Tremblay MÈ (2023). Emerging epigenetic dynamics in gut-microglia brain axis: experimental and clinical implications for accelerated brain aging in schizophrenia. Front Cell Neurosci.

[128] Jabin N, Rahman MM, Salam MT (2023). Cohort profile: Bangladesh Cook Stove Pregnancy Cohort Study (CSPCS). BMJ Open.

[129] Li S, Guo B, Dong K (2023). Association of long-term exposure to ambient PM(2.5) and its constituents with gut microbiota: evidence from a China cohort. Sci Total Environ.

[130] Nair B, Kamath AJ, Tergaonkar V, Sethi G, Nath LR (2024). Mast cells and the gut-liver axis: implications for liver disease progression and therapy. Life Sci.

[131] Di Renzo L, Gualtieri P, Frank G (2024). Exploring the exposome spectrum: unveiling endogenous and exogenous factors in non-communicable chronic diseases. Diseases.

[132] Bhardwaj G, Riadi Y, Afzal M (2024). The hidden threat: environmental toxins and their effects on gut microbiota. Pathol Res Pract.

[133] Dosh L, Ghazi M, Haddad K (2024). Probiotics, gut microbiome, and cardiovascular diseases: an update. Transpl Immunol.

[134] Chang C, Gupta R, Sedighian F (2024). Subchronic inhalation exposure to ultrafine particulate matter alters the intestinal microbiome in various mouse models. Environ Res.

[135] Zhang M, Liang C, Chen X, Cai Y, Cui L (2024). Interplay between microglia and environmental risk factors in Alzheimer's disease. Neural Regen Res.

[136] Zhang K, Paul K, Jacobs JP, Cockburn MG, Bronstein JM, Del Rosario I, Ritz B (2024). Ambient long-term exposure to organophosphorus pesticides and the human gut microbiome: an observational study. Environ Health.

[137] Belloumi D, García-Rebollar P, Calvet S (2024). Impact of including two types of destoned olive cakes in pigs' diets on fecal bacterial composition and study of the relationship between fecal microbiota, feed efficiency, gut fermentation, and gaseous emissions. Front Microbiol.

[138] Dehghani A, Wang L, Garssen J (2024). Synbiotics, a promising approach for alleviating exacerbated allergic airway immune responses in offspring of a preclinical murine pollution model. Environ Toxicol Pharmacol.

[139] Dahiya P, Kumari S, Behl M (2024). Guardians of the gut: harnessing the power of probiotic microbiota and their exopolysaccharides to mitigate heavy metal toxicity in humans for better health. Probiotics Antimicrob Proteins.

[140] Campolim CM, Schimenes BC, Veras MM, Kim YB, Prada PO (2024). Air pollution accelerates the development of obesity and Alzheimer's disease: the role of leptin and inflammation - a mini-review. Front Immunol.

[141] Utembe W, Kamng'ona AW (2024). Inhalation exposure to chemicals, microbiota dysbiosis and adverse effects on humans. Sci Total Environ.

[142] Cruells A, Cabrera-Rubio R, Bustamante M (2024). The influence of pre- and postnatal exposure to air pollution and green spaces on infant's gut microbiota: results from the MAMI birth cohort study. Environ Res.

[143] Mazumder MH, Hussain S (2024). Air-pollution-mediated microbial dysbiosis in health and disease: lung-gut axis and beyond. J Xenobiot.

[144] Dai S, Wang Z, Cai M, Guo T, Mao S, Yang Y (2024). A multi-omics investigation of the lung injury induced by PM(2.5) at environmental levels via the lung-gut axis. Sci Total Environ.

[145] Khandayataray P, Murthy MK (2024). Dietary interventions in mitigating the impact of environmental pollutants on Alzheimer's disease - a review. Neuroscience.

[146] Wong CF, Saif UM, Chow KL (2024). Applications of charcoal, activated charcoal, and biochar in aquaculture - a review. Sci Total Environ.

[147] Liu CX, Liu YB, Peng Y, Peng J, Ma QL (2024). Causal effect of air pollution on the risk of cardiovascular and metabolic diseases and potential mediation by gut microbiota. Sci Total Environ.

[148] Hameed S, Karim N, Wasay M, Venketasubramanian N (2024). Emerging stroke risk factors: a focus on infectious and environmental determinants. J Cardiovasc Dev Dis.

[149] Keerthy D, Spratlen MJ, Wen L (2024). An evaluation of in utero polycyclic aromatic hydrocarbon exposure on the neonatal meconium microbiome. Environ Res.

[150] Qiu T, Fang Q, Zeng X (2024). Short-term exposures to PM(2.5), PM(2.5) chemical components, and antenatal depression: exploring the mediating roles of gut microbiota and fecal short-chain fatty acids. Ecotoxicol Environ Saf.

[151] Du Y, Wang Q, Zheng Z (2024). Gut microbiota influence on lung cancer risk through blood metabolite mediation: from a comprehensive Mendelian randomization analysis and genetic analysis. Front Nutr.

[152] Li C, Chen H, Gu Y (2024). Causal effects of PM(2.5) exposure on neuropsychiatric disorders and the mediation via gut microbiota: a Mendelian randomization study. Ecotoxicol Environ Saf.

[153] Zha H, Xia J, Wang K, Xu L, Chang K, Li L (2024). Foodborne and airborne polyethersulfone nanoplastics respectively induce liver and lung injury in mice: comparison with microplastics. Environ Int.

[154] Chen CM, Yang YS, Chou HC (2024). Maternal diesel particle exposure alters gut microbiota and induces lung injury in rat offspring. Ecotoxicol Environ Saf.

[155] Padhi P, Zenitsky G, Jin H (2024). Environmental chemical-induced adverse effects on gut microbiota and their implications for the etiopathogenesis of chronic neurological diseases. Advances in Neurotoxicology.

[156] Guilloteau E, Coll P, Lu Z (2022). Murine in utero exposure to simulated complex urban air pollution disturbs offspring gut maturation and microbiota during intestinal suckling-to-weaning transition in a sex-dependent manner. Part Fibre Toxicol.

[157] Santos-Silva L, Roque WF, de Moura JM (2024). Toxic metals in Amazonian soil modify the bacterial community associated with Diplopoda. Sci Total Environ.

[158] Gouider R, Souissi A, Mrabet S, Gharbi A, Abida Y, Kacem I, Gargouri-Berrechid A (2024). Environmental factors related to multiple sclerosis progression. J Neurol Sci.

[159] Pan R, Yi X, Xu Y (2024). Association between indoor PM(2.5) components and accelerated biological aging in schizophrenia patients: evidence from multi-omics mechanisms. J Hazard Mater.

[160] Shao W, Pan B, Li Z (2024). Gut microbiota mediates ambient PM(2.5) exposure-induced abnormal glucose metabolism via short-chain fatty acids. J Hazard Mater.

[161] Sugden SG, Merlo G (2024). What do climate change, nutrition, and the environment have to do with mental health?. Am J Lifestyle Med.

[162] Zaltman C, do Espírito Santo PA, de Magalhães Costa MH (2024). Ambient air pollution and inflammatory bowel disease - a narrative review. Dig Med Res.

[163] Wang Z, Xu M, Li Q, Lu S, Liu Z (2025). Subchronic chloroform exposure causes intestinal damage and induces gut microbiota disruption and metabolic dysregulation in mice. Environ Toxicol.

